# A novel functional *IKBKE* variant activating NFAT in a patient with polyarthritis and a remittent fever

**DOI:** 10.3389/fimmu.2024.1475179

**Published:** 2024-10-25

**Authors:** Saeko Yamada, Yasuo Nagafuchi, Mamiko Yamada, Hisato Suzuki, Bunki Natsumoto, Mineto Ota, Ikuo Takazawa, Hiroaki Hatano, Masanori Kono, Hiroaki Harada, Hirofumi Shoda, Tomohisa Okamura, Kenjiro Kosaki, Keishi Fujio

**Affiliations:** ^1^ Department of Allergy and Rheumatology, Graduate School of Medicine, The University of Tokyo, Tokyo, Japan; ^2^ Department of Functional Genomics and Immunological Diseases, Graduate School of Medicine, The University of Tokyo, Tokyo, Japan; ^3^ Center for Medical Genetics, Keio University School of Medicine, Tokyo, Japan; ^4^ Department of Medical Genetics, Institute of Medicine, University of Tsukuba, Ibaraki, Japan

**Keywords:** *IKBKE*, the inhibitor of κB kinase ϵ (IKKϵ), rare variant, T cell, type I IFN, NFκB

## Abstract

**Background:**

*IKBKE* is a negative regulator of T cell activation and one of the key activators of type I interferon (IFN) and NFκB signaling via non-classical pathways. The upstream single nucleotide polymorphism of *IKBKE* (rs2297550-G) is a genome-wide association study risk variant of systemic lupus erythematosus, and is associated with decreased *IKBKE* expression in T cells by expression quantitative trait locus analysis.

**Case presentation:**

A 48-year-old female had a remittent fever, arthritis, and oral ulcers for 20 years. She had a poor response to corticosteroids or disease-modifying antirheumatic drugs, including the tumor necrosis factor-α antagonist, etanercept, and the anti-interleukin-6 receptor antibody, tocilizumab.

**Method:**

She participated in the Initiative on Rare and Undiagnosed Disease (IRUD), and whole-exome sequencing (WES) was performed. Functional analyses were conducted by transfecting the identified variants into reporter cells to assess the activation of NFAT and NFκB signaling. Additionally, peripheral blood RNA- sequencing (RNA-seq) data were compared with those from healthy individuals to evaluate the gene expression profiles of immune cells.

**Result:**

WES identified a novel heterozygous c.1877G>A, p(Cys626Tyr) variant in *IKBKE*. Functional analysis indicated that this variant led to increased activity of NFAT (*p* = 0.015) and decreased activity of NFκB and type I IFN (*p* = 0.00068 and 0.00044, respectively). The patient had a remarkably low proportion of Naïve CD4 T cells. RNA-seq of peripheral blood immune cell subsets revealed significant differences in gene expression, especially in T cells.

**Conclusion:**

A novel functional heterozygous variant in *IKBKE* is described in a patient with a remittent fever and arthritis. The data suggest that *IKBKE* is an important negative regulator of inflammation, particularly in T cells, and this *IKBKE* variant might be the underlying cause of a novel autoinflammatory pathology.

## 1 Introduction

*IKBKE*, which encodes the inhibitor of κB kinase (IKK) ϵ (IKKϵ, also known as IKKi), enhances IKKϵ expression in T cells and promotes phosphorylation of NFATc1 while suppressing T cell responses. Therefore, IKKϵ serves as a crucial negative regulator of T cell activation and loss of IKKϵ function can lead to inflammation (1).

Furthermore, IKKϵ activates NFκB, interferon (IFN), and signal transducer and activator of transcription signaling (2–5). IKKϵ is essential for regulating antiviral signaling pathways. IKKϵ is located downstream of virus sensors and Toll-like receptors (TLRs) and upstream of interferon regulatory factors (IRFs). Tumor necrosis factor (TNF)-α and interleukin (IL)-1β induce IKK activation through different pathways involving polyubiquitination (6, 7). Activated IKKϵ, along with its homolog, TANK-binding kinase 1 (TBK1), has a crucial role in phosphorylating NFκB inhibitors, leading to dissociation of the inhibitor/NFκB complex and subsequent degradation of the inhibitor (8). IKKϵ also activates IRF3 in conjunction with activated NFκB, resulting in upregulation of type I IFN (2, 9).

Patients presenting with atypical fever and systemic symptoms are often observed, yet due to the frequent unknown etiology, they face significant unmet medical needs and are unable to receive appropriate treatment. The pathogenesis of rare hereditary autoinflammatory diseases, such as familial Mediterranean fever, cryopyrin-associated periodic syndrome, and A20 haploinsufficiency, has been elucidated through genetic analysis, leading to advances in treatment (10–13). However, there are still numerous cases classified as undiagnosed atypical diseases for which the cause remains unknown. The Initiative on Rare and Undiagnosed Disease (IRUD) is a nationwide research consortium in Japan aimed at genetic diagnosis and elucidation of the pathogenesis of such rare and atypical undiagnosed diseases (14). Whole-exome sequencing (WES) has identified variants that may serve as candidate causes of diseases, resulting in the creation of rich databases and the proposal of numerous new disease concepts (15).

Herein a woman with a novel functional variant in *IKBKE*, who had been experiencing polyarthritis and remittent fever for 20 years, is described. Functional analysis identified the variant activating T cells, while also diminishing NFκB and type I IFN signaling. The patient exhibited a notably diminished proportion of Naïve CD4 T cells. RNA sequencing (RNA-seq) of immune cell subsets from peripheral blood revealed diverse immune cell gene expression changes, especially in T cells. This case suggests the possibility of a novel autoinflammatory disorder due to impaired function of *IKBKE* and T cell activation.

## 2 Case description

The patient was a 48-year-old Japanese female who had been experiencing polyarthralgias, recurrent fevers lasting 1-2 weeks every 1-2 months, and mouth ulcers since 28 years of age. Her symptoms did not improve following a bilateral tonsillectomy. At 43 years of age, 10 mg/day of prednisolone (PSL) was initiated due to suspected rheumatoid arthritis (RA) or Behçet’s syndrome based on oral and genital ulcers, folliculitis, arthritis, and positivity for HLA-A26, which is positive in approximately 30% of patients with Behçet’s disease (16). However, her fever worsened when the PSL dose was reduced to 5 mg/day. At 45 years of age, the PSL dose was increased to 30 mg/day based on a suspicion of TNF receptor-associated periodic syndrome but her symptoms recurred upon reducing the PSL dose to 5 mg/day. Disease-modifying antirheumatic drugs, including iguratimod, which inhibits NF-κB activation, salazosulfapyridine, the TNF-α antagonist, etanercept, and the anti-IL-6 receptor antibody, tocilizumab, were ineffective for the arthritis. She was diagnosed with asthma at another hospital at 47 years of age due to dyspnea and wheezing; however, her respiratory function tests were normal, there was no increase in total IgE levels, and a CT scan after transferring to our hospital showed no notable findings in the lung fields. Laboratory findings revealed an elevated white blood cell count (10,400/µl [neutrophils, 74.6%; lymphocytes, 19.7%; monocytes, 4.9%; eosinophils, 0.6%; and basophils, 0.2%], normal range [NR] <8600/µl) and an elevated erythrocyte sedimentation rate (42 mm/h; NR <15 mm/h) under immune suppression. The serum C-reactive protein level was within the NR (0.06 mg/dl, NR <0.3 mg/dl). The serum IgM levels were within the NR, but the IgG and IgA levels were low (IgG, 562 mg/dl; IgA, 39 mg/dl; and IgM, 52 mg/dl). All measured autoantibodies, including antinuclear antibodies, anti-citrullinated protein/peptide antibody, and rheumatoid factor, were negative.

At 48 years of age, due to an atypical clinical presentation, she participated in IRUD and WES was performed to search for candidate causal variants that accounted for the symptoms. Unfortunately, due to estrangement from all blood relatives, including both parents, blood sampling from the patient’s blood relatives was not possible. However, according to the patient, there were no individuals among the blood relatives who exhibited similar symptoms. At the same time, peripheral blood immune cell RNA-seq was also performed to comprehensively compare the proportion and gene expression of immune cell subsets to the Immune Cell Gene Expression Atlas from the University of Tokyo (ImmuNexUT) dataset (17), which is an extensive catalog of immune cell gene expression that we recently created. Subsequently, treatment with disease-modifying antirheumatic drugs was continued but self-reported symptoms, such as joint pain and fevers, remained unchanged.

At 53 years of age, she was found unconscious and pronounced dead. *Klebsiella pneumoniae* was detected in blood cultures after her death.

**Figure 1:**
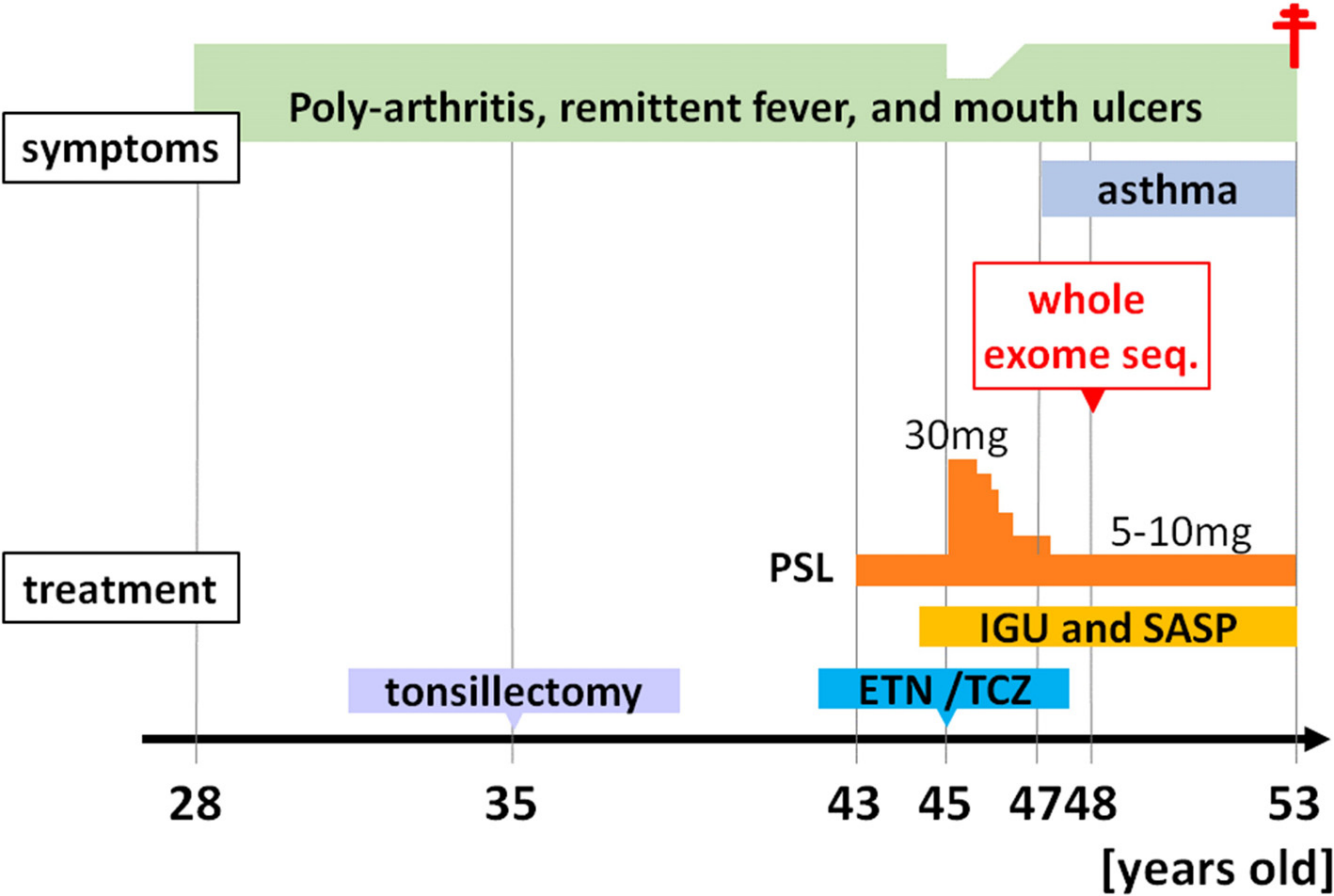
Clinical course of the patient. Seq, sequencing; PSL, prednisolone; IGU, iguratimod; SASP, salazosulfapyridine; ETN, etanercept; TCZ, tocilizumab.

The progression of her condition is illustrated in **Figure 1**.

## 3 Methods

### 3.1 Identification of genetic variants

This study was approved by the Ethics Committees of the University of Tokyo (G10109). Written informed consent was obtained from each subject in accordance with the Declaration of Helsinki. An exome analysis was performed as previously reported (18). Genomic DNA was extracted from peripheral blood samples from the patient. Exome sequencing was performed using the SureSelectXT Human All V6 (Agilent Technologies, Santa Clara, CA, USA) and the HiSeq platform (Illumina, San Diego, CA, USA). The sequence reads were mapped to the human reference genome (GRCh37) according to the best-practice guidelines for Burrows-Wheeler Aligner [BWA] (19) and the Genome Analysis Tool Kit [GATK] (20) and the integrated analysis suite variant tools (21). The filtered variants were annotated with SnpEff (22).

### 3.2 Functional analysis experiment of *IKBKE* variants

Jurkat-Lucia™ nuclear factor of activated T cells (NFAT) cells (Jurkat cells capable of detecting NFAT activation) were transfected with plasmids containing wild-type *IKBKE* and *IKBKE* variants. Note that *IKBKE* is not knocked out in these cells. After a period of cultivation, the transfected cells were collected using flow cytometry. Subsequently, the sorted cells were stimulated with CD3 and CD28, and the activation of NFAT was quantitatively evaluated using a commercial kit (QUANTI-Luc; InvivoGen, San Diego, CA, USA).

Additionally, plasmids containing wild-type (*IKBKE*) and *IKBKE* variants were transfected into two types of reporter cells: TNFα reporter HEK293 cells, which monitor NFκB signaling activation, and type I IFN reporter HEK293 cells. Note that *IKBKE* is not knocked out in these cells. After 48 h of culture, the supernatant was collected and the levels of secreted NFκB and type I IFN were quantitatively evaluated by measuring the amount of secreted alkaline phosphatase (SEAP) produced using a commercially available kit (QUANTI-blue; InvivoGen, San Diego, CA, USA).

A detailed description is included in the **Supplementary Data Sheet 1**.

### 3.3 Comparison of the peripheral blood immune cell proportion and RNA-seq data with healthy individuals

This study was approved by the Ethics Committees of the University of Tokyo (G10095). Twenty-six peripheral blood immune cell subsets were isolated from the patient and RNA-seq was performed using the identical experimental and analytic pipelines of the ImmuNexUT dataset (17). The definitions of each of these cell subsets, which were based on cell surface antigens, are provided in **Supplementary Table S1**. RNA-seq data from the peripheral blood of 64 healthy controls (HCs) available in the ImmuNexUT dataset (17) were used as controls. The methodology for RNA-seq is detailed in the **Supplementary Data Sheet 1**. The cell proportions between the case and HCs were compared. Differences in gene expression using edgeR and Reactome pathway analysis were also compared.

### 3.4 eQTL analysis of SLE risk variant

The rs2297550-G single nucleotide polymorphism (SNP) located upstream of *IKBKE* is known to be a risk locus for systemic lupus erythematosus (SLE) in genome-wide association studies (GWAS) (23, 24). An analysis of cis-expression quantitative trait loci (eQTL) effects on *IKBKE* for each cell subset in the publicly available data of 416 individuals from ImmuNexUT dataset was performed for this allele (17).

## 4 Results

### 4.1 Identification of a novel *IKBKE* variant through exome sequencing

**Figure 2:**
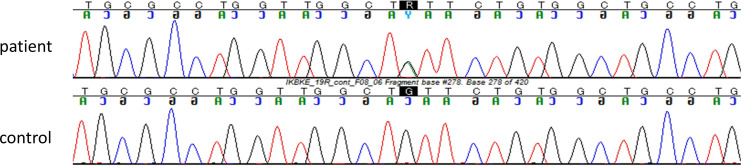
The variant c.1877G>A in *IKBKE* gene was confirmed by Sanger sequencing. The Sanger sequencing confirmed that the patient had a heterozygous variant of the *IKBKE* gene [Chr1(GRCh37):g.206666397G>A, NM_014002.3:c.1877G>A, p.(Cys626Tyr)].

Detailed results of filtering candidate causal single nucleotide variants in the patient are shown in **Supplementary Figure S1**. The patient had a heterozygous variant in exon 19 of the *IKBKE* gene [Chr1(GRCh37):g.206666397G>A, NM_014002.3:c.1877G>A, p.(Cys626Tyr)]. This variant was confirmed by Sanger sequencing (**Figure 2**) and is extremely rare. Specifically, c.1877G>A was not encountered in any of a 54,302-person cohort of normal Japanese individuals (25) and in the gnomAD database (26), which is the largest database globally but includes less than 10% participation from East Asian individuals. This variant was also absent in pathogenic variant databases, such as the ClinVar and HGMD databases (27, 28).

The pathogenicity of the variant was predicted as follows: Polyphen2 HumDiv/Var predicted the variant to be benign (29); MutationTaster (v2013) predicted the variant to be benign supporting (30); and the combined annotation-dependent depletion (CADD) score of 22 corresponded to a deleterious result (31). Overall, the allele (c.1877G>A) was scored as a “variance of uncertain significance (VUS)” (PM6 and BP6) according to the standards and guidelines for the interpretation of sequence variants by the American College of Medical Genetics and Genomics (32). The *IKBKE* gene has not been established as a human disease candidate gene, but the rs2297550-G SNP located upstream of *IKBKE* is known as a risk locus for SLE, as previously reported (23, 24).

No other pathogenic variant in genes known to be causative for periodic fever syndromes was identified. In addition to the *IKBKE* gene, the *WFS1* gene has been identified as a gene associated with “autoimmune diseases,” as defined by The Human Gene Mutation Database. The *WFS1* gene, however, which is responsible for Wolfram-like syndrome and deafness, cannot explain the patients’ symptoms (**Supplementary Figure S1**). No other candidate disease-causing genes with variants in autosomal dominant or recessive models that could explain the patient’s symptoms were identified, except for the *IKBKE* gene.

### 4.2 Functional analysis experiment of the C626Y *IKBKE* variant

**Figure 3:**
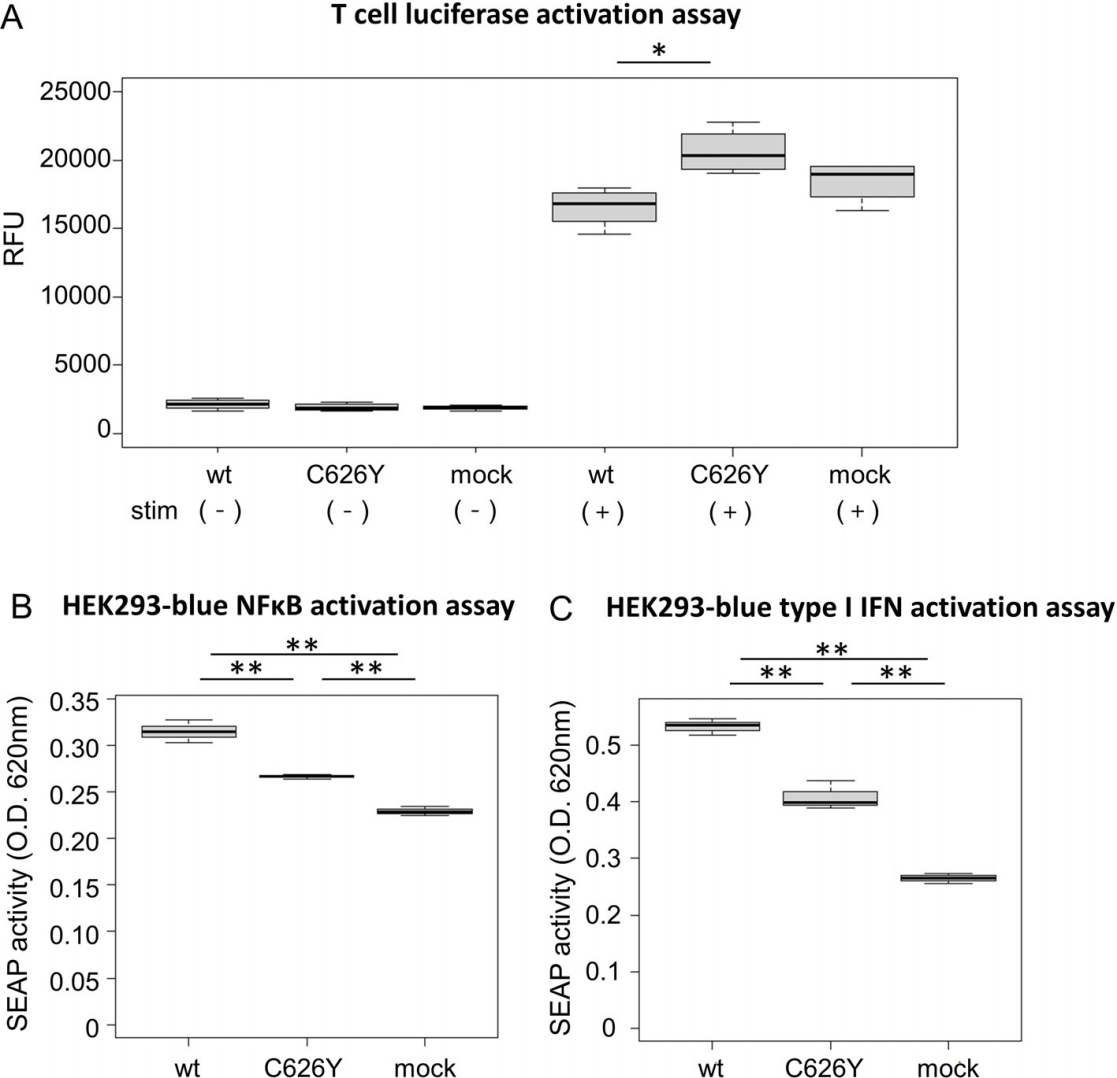
Functional experiments of *IKBKE* variant transfected into reporter cells. **(A)** Jurkat-Lucia NFAT cells (Jurkat cells capable of detecting NFAT activation) were transfected with plasmids containing wild-type *IKBKE* or mutant *IKBKE.* After a certain period of cultivation, the transfected cells were collected using flow cytometry. After sorting, the NFAT activation levels of each cell under unstimulated conditions were shown on the left side of the figure, and the NFAT activation levels after CD3 and CD28 stimulation were shown on the right side of the figure. **(B, C)** NFκB and type I IFN activation was observed from HEK 293 reporter cells, [**(B)** Human TNF-α SEAP reporter HEK293 cells, **(C)** Human HEK293 cells - Type I IFNs reporter cells] transfected with plasmids containing wild-type *IKBKE* or mutant *IKBKE*. The activation of NFκB and type I IFN were quantitatively evaluated in the supernatant by measuring the amount of SEAP produced along with them in the reporter cells. **(A)** was experimented in quadruplicate, while **(B, C)** were experimented in triplicate. Pairwise comparisons using t-test with Bonferroni adjustment. IFN, interferon; TNF, tumor necrosis factor; SEAP, secreted alkaline phosphatase; wt. wild-type; O.D., optical density; RFU, relative fluorescence unit. *p < 0.05, ** *p* < 0.005.

To investigate the impact of the novel variant on T cells, wild-type *IKBKE* and the C626Y *IKBKE* variants were transfected into Jurkat-Lucia™ NFAT cells. Under unstimulated conditions, there was no significant difference in NFAT activation between the group transfected with wild-type *IKBKE* and the group transfected with the *IKBKE* variant (*p* > 0.99; **Figure 3A**, left). However, upon stimulation with CD3 and CD28, the group transfected with the *IKBKE* variant had a significantly higher level of NFAT production compared to the group transfected with wild-type *IKBKE* (*p* = 0.015; **Figure 3A**, right).

Transfection of wild-type *IKBKE* and the C626Y *IKBKE* variant into two types of HEK293 cells demonstrated that the *IKBKE* variant in the patient significantly reduced NFκB and type I IFN activity compared to the wild-type variant (*p* = 0.00068 and 0.00044, respectively; **Figures 3B, C**). The C626Y *IKBKE* variant is situated within exon 19, precisely at the 1877^th^ base pair (corresponding to the 626^th^ amino acid). This alteration in structure could impede the formation of IKKϵ homodimers, binding affinity to TBK1, and interaction with adaptor proteins. Consequently, this alteration might diminish the activity of IKKϵ, resulting in a decreased signaling cascade of NFκB and type I IFN.

### 4.3 Dysregulated immune cell proportion and gene expression in the case

**Figure 4:**
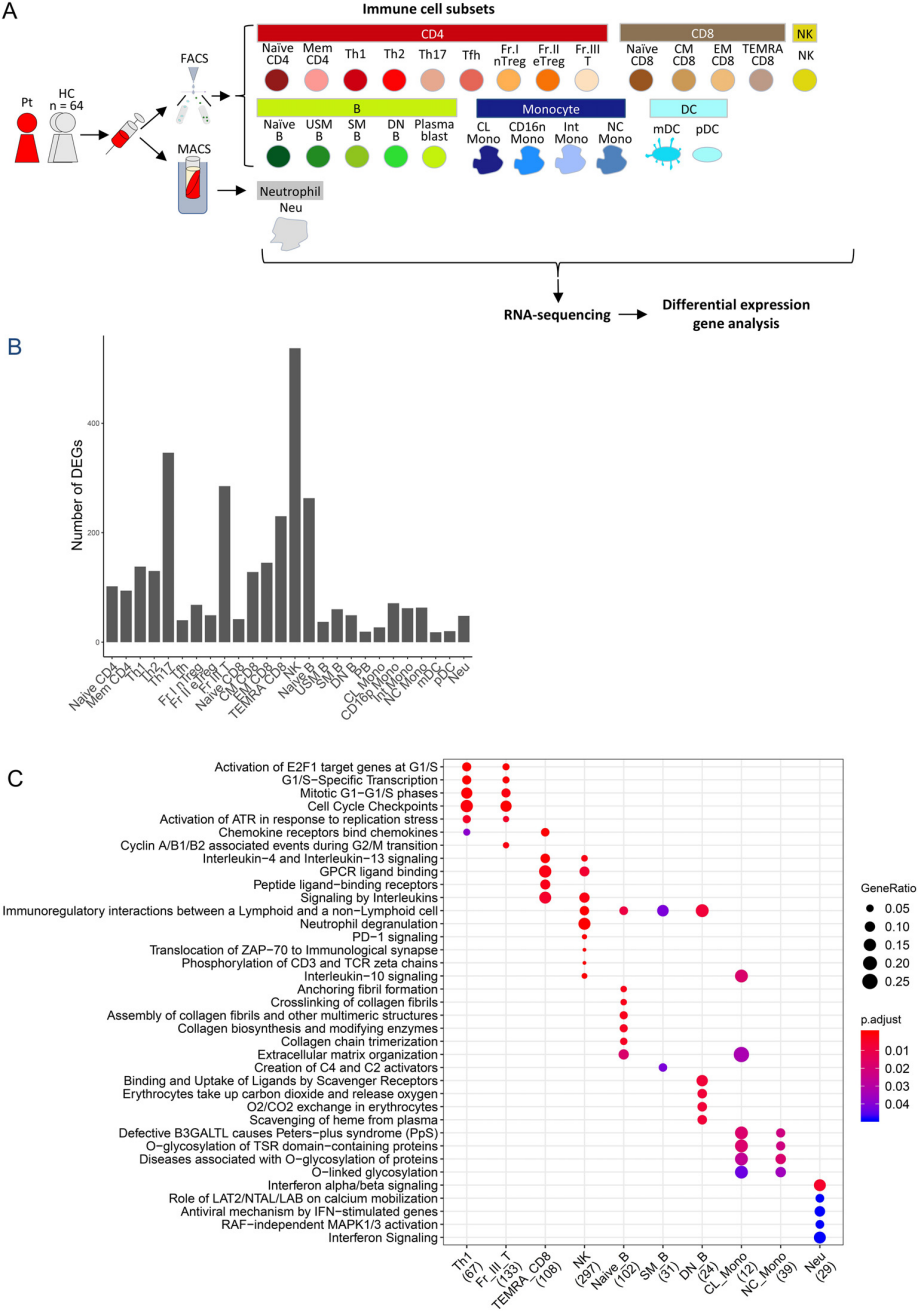
Peripheral blood immune-cell RNA-seq analysis of the case. **(A)** Flowchart illustrating the process of cell sorting, RNA sequencing, and analysis. **(B)** Number of differentially-expressed genes. **(C)** Reactome pathway analysis of differentially expression genes. Pt, patient; HC, healthy controls; FACS, fluorescence assisted cell sorting; MACS, magnetic-activated cell sorting, Naïve CD4, Naïve CD4 T cells; Mem CD4, Memory CD4 T cells; Th1, T helper 1 cells; Th2, T helper 2 cells; Th17, T helper 17 cells; Tfh, T follicular helper cells; Fr. I nTreg, Fraction I naïve regulatory T cells; Fr. II eTreg, Fraction II effector regulatory T cells; Fr. III T, Fraction III non-regulatory T cells; Naïve CD8, Naïve CD8 T cells; CM CD8, Central memory CD8 T cells; EM CD8, Effector memory CD8 T cells; TEMRA CD8, CD8+ T effector memory CD45RA+ cells; NK,Natural killer cells; Naïve B, Naïve B cells; USM B, Unswitched memory B cells; SM B, Switched memory B cells; DN B, Double negative B cells; Plasmablast, Plasmablasts; CL Mono, Classical monocytes; CD16p Mono, CD16 positive monocytes, Int Mono, Intermediate monocytes; NC Mono, Non-classical monocytes; mDC, Myeloid dendritic cells; pDC, Plasmacytoid dendritic cells.

To comprehensively investigate the proportion and transcriptomic differences in immune cell populations between the patient and HCs, RNA-seq was performed on 26 peripheral blood immune cell subsets and differential gene expression was analyzed (**Figure 4A**). The HC group consisted of 64 individuals from the ImmuNexUT database, which was comprised of 16 males and 48 females with an average age of 47.2 ± 15.0 years. Compared to the HCs, the patient had a remarkably low Naïve CD4 T cell proportion within the CD4 T cells (**Table 1**). In addition, an increased proportion of classical monocytes (CL Mono) and a decreased proportion of non-classical monocytes (NC Mono) were noted within the CD3^-^CD19^-^/HLA-DR^+^/CD56^-^ lymphocytes (**Table 1**).

**Table 1:** Proportions of subsets in the patient, compared with healthy controls.

Parent	Subset	Percentage per parent [%] (IQR)		Z score
		Patient	HC (n=64)	
CD4	Naïve CD4	10.2	36.69 (30.76-45.33)	-2.11 *
	Mem CD4	44.54	48.36 (41.45-55.72)	-0.32
	Th1	4.56	3.01 (2.07-3.94)	1.16
	Th2	2.57	2.91 (1.79-3.85)	-0.23
	Th17	3.8	2.56 (1.82-3.20)	1.12
	Tfh	8.25	11.40 (8.56-13.72)	-0.8
	Fr.I nTreg	0.85	2.46 (1.53-2.78)	-1.28
	Fr.II eTreg	0.71	1.08 (0.76-1.35)	-0.78
	Fr.III T	5.48	6.03 (3.89-7.68)	-0.2
CD8	Naïve CD8	18.6	34.97 (17.83-47.54)	-0.84
	CM CD8	4.18	4.29 (2.39-6.04)	-0.04
	EM CD8	26.11	18.03 (12.90-22.02)	0.9
	TEMRA CD8	1.4	13.84 (5.82-19.14)	-1.15
Lymphocytes	NK	6.24	14.04 (9.68-18.43)	-1.19
B	Naïve B	50.36	64.10 (57.30-75.45)	-0.9
	USM B	20.64	12.14 (6.67-15.78)	1
	SM B	11.38	16.52 (11.44-19.94)	-0.48
	DN B	3.15	3.31 (1.93-3.42)	-0.04
	Plasmablast	0.39	2.25 (0.84-2.90)	-0.81
CD3^-^CD19^-^/HLA-DR^+^/CD56^-^ myeloid cells	CL Mono	93.59	78.86 (77.09-83.74)	2.84 *
	Int Mono	1.11	2.92 (2.02-3.46)	-1.34
	NC Mono	0.84	8.59 (6.02-10.60)	-2.12 *
	mDC	1.1	2.47 (1.77-2.99)	-1.57
	pDC	0.31	1.47 (0.94-1.59)	-1.3

Gating strategy is summarized in **Supplementary Table S1**. Because some of the cells were gated with overlap (for example, Memory CD4 and Th1), and some borderline cells were excluded in the gating strategy (17), the sum of the cell subsets does not align with 100%.

IQR, interquartile range; HC, healthy control.

Data of CD16p Mono was unavailable.

* |z score| > 1.96.

Widespread differential immune cell gene expression was also demonstrated in the patient (**Figure 4B**). In T cells, particularly in Th17, a subtype of effector T cells, there were numerous differentially-expressed genes (DEGs) (**Figure 4B**). In TEMRA CD8 T cells, in which DEGs were abundant (**Figure 4B**), noticeable alterations were observed in the G protein-coupled receptor (GPCR) ligand binding pathway and interleukin signaling in Reactome pathway analysis (**Figure 4C**), along with high expression of chemokine receptor genes and *MEF2C*, which controls downregulation of CXCR5 promoter activity (33) (**Supplementary Table S2A**). Thus, activation of T cells was indicated. In NK cells, the highest number of DEGs was observed (**Figure 4B**), along with variations in pathways, such as the GPCR ligand binding pathway and signaling by interleukins (**Figure 4C**). Additionally, in NK cells, characteristic high expression of inflammasome-related genes, such as *MEFV* and *NLRP3*, as well as interleukin-related genes, including *IL1B*, was detected (**Supplementary Table S2B**), suggesting activation of NK cells. In neutrophils, significant alterations were observed in the type I IFN pathway (**Figure 4C**), with actual downregulation of downstream genes of type I IFN, such as *OAS2*, *OAS1*, and *OAS3* (**Supplementary Table S2C**).

### 4.4 eQTL analysis of the SLE risk *IKBKE* variant

**Figure 5:**
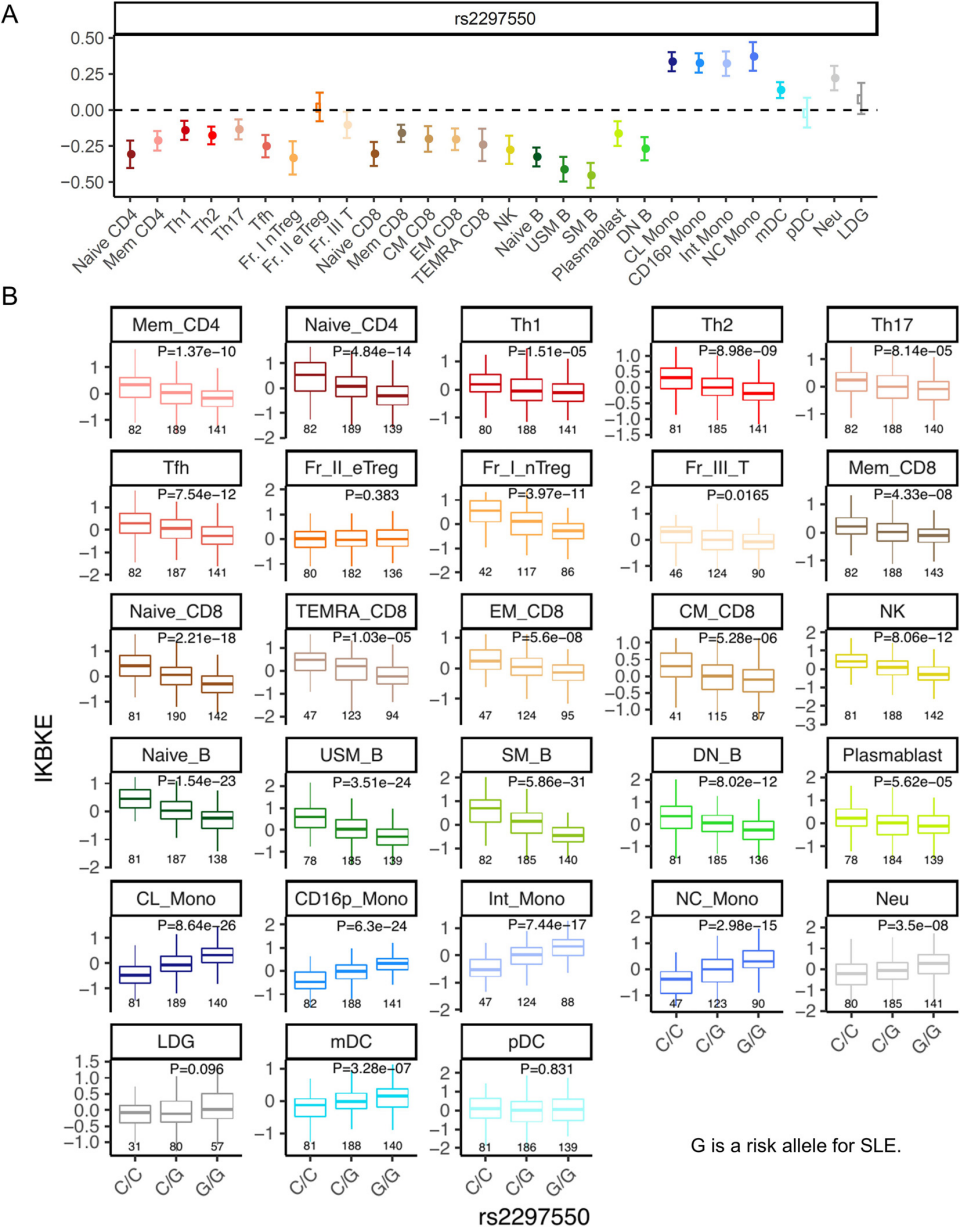
eQTL analysis of the SLE risk *IKBKE* variant. **(A)** The cis-eQTL effect beta by this allele. A positive beta corresponds to an increase in gene expression due to the risk allele. The error bars represent the 95% confidence interval. **(B)** cis-eQTL effects boxplots on the expression of *IKBKE* across cell subsets. G is a risk allele for systemic lupus erythematosus. The *p*-value represents the association of eQTL (linear regression) *p*-values. Below the boxplot are the sample sizes for each group. Naïve CD4, Naïve CD4 T cells; Mem CD4, Memory CD4 T cells; Th1, T helper 1 cells; Th2, T helper 2 cells; Th17, T helper 17 cells; Tfh, T follicular helper cells; Fr. I nTreg, Fraction I naïve regulatory T cells; Fr. II eTreg, Fraction II effector regulatory T cells; Fr. III T, Fraction III non-regulatory T cells; Naïve CD8, Naïve CD8 T cells; CM CD8, Central memory CD8 T cells; EM CD8, Effector memory CD8 T cells; TEMRA CD8, CD8+ T effector memory CD45RA+ cells; NK,Natural killer cells; Naïve B, Naïve B cells; USM B, Unswitched memory B cells; SM B, Switched memory B cells; DN B, Double negative B cells; Plasmablast, Plasmablasts; CL Mono, Classical monocytes; CD16p Mono, CD16 positive monocytes, Int Mono, Intermediate monocytes; NC Mono, Non-classical monocytes; mDC, Myeloid dendritic cells; pDC, Plasmacytoid dendritic cells.

The rs2297550-G SNP located upstream of *IKBKE* has been reported as a risk allele for SLE in GWAS (23, 24). This allele is associated with increased expression of *IKBKE* in monocytes and decreased expression of *IKBKE* in T cells, IFN-stimulated monocytes, B cells, and NK cells, as indicated by eQTL analysis (24). Indeed, analysis of cis-eQTL effects in the ImmuNexUT dataset (17) revealed that this allele suppressed the expression of *IKBKE* across most subsets in T, B, and NK cells, except for Fr. II effector Treg, in which no eQTL effect was observed (**Figure 5**). Additionally, an upregulation of *IKBKE* expression due to this allele was observed in monocytes, neutrophils, and mDCs. Because eQTL analysis exclusively focuses on common variants, it is not possible to estimate the function of the novel C626Y *IKBKE* variant identified in this patient. However, the finding that the SLE risk polymorphism reduced the expression of *IKBKE* in T cells is consistent with the results that the C626Y *IKBKE* variant increased the activity of NFAT in T cells.

## 5 Discussion

The focus of this study involved a patient with a history of polyarthritis and recurrent fevers for 20 years. The novel functional C626Y variant in her *IKBKE* gene was investigated. IKKϵ is known to function as a crucial negative regulator of T cell activation (1). Indeed, functional analysis revealed that this variant led to increased NFAT activity in T cells (**Figure 3A**). Comparison of the RNA-seq data of immune cell subsets from peripheral blood of this patient with HCs suggested a decrease in the number of Naive T cells (**Table 1**) and activation of effector T cells, such as Th17 and TEMRA CD8 T cells (**Figures 4B, C**). Altogether, these results suggest that the C626Y *IKBKE* variants underlie the immunologic and clinical abnormalities of the patient. This case suggests the possibility of novel inflammatory diseases due to impaired function of *IKBKE* and T cell activation.

Functional analysis of the *IKBKE* variant in cultured T cell lines revealed its potential role in T cell activation (**Figure 3A**). Furthermore, to comprehensively characterize this case, WES, comprehensive immune cells subset ratio analysis, and gene expression analysis were performed (**Figure 4**). Through this unique integrated approach, a reduction in Naive T cells and activation of T cells were identified as the possible pathophysiologic alteration. This case suggests that ‘deep immune cell phenotyping’ of atypical cases suspected of immune-inflammatory diseases could provide valuable insight for understanding the underlying pathophysiology.

In T cells, IKKϵ has a crucial role as a negative regulator of T cell activation and is considered a potential target for immunotherapy (1). In the case presented the variant appeared to release this negative regulatory mechanism, as suggested by the activation of T cells. IKKϵ also modulates IL-17 signaling and contributes to the maintenance and/or proliferation of Th17 cells through the glycogen synthase kinase (GSK)-AKT-mammalian target of rapamycin (mTOR) pathway (34–36).

Intriguingly, analysis of cis-eQTL effects of the SLE risk rs2297550-G SNP located upstream of *IKBKE* did not show an eQTL effect suppressing *IKBKE* expression specifically in Fr. II eTreg among T cells (**Figure 5**). Therefore, in the pathogenesis of SLE, the activation of effector T cells due to a decrease in *IKBKE* is thought to be important. There is also the possibility that a decrease in *IKBKE* expression in B cells, along with an increase in *IKBKE* in monocytes and neutrophils or eQTLs in tissues may be associated with the risk of developing SLE. Therefore, elucidating the immunologic functions of the *IKBKE* gene in the pathogenesis solely through eQTL analysis may be challenging.

Overall, considering the two roles of *IKBKE*—1) suppressing T cell activation and 2) promoting IFN production—it appears that both functions are impaired in the variant observed in this case. This may explain why the clinical presentation leans towards an autoinflammatory disease with a less prominent IFN signature. On the other hand, in the population with the rs2297550-G risk allele in SLE, T cells may become activated due to low *IKBKE* expression (1), while in myeloid cells, *IKBKE* expression is elevated (**Figure 5**). This could lead to increased type I IFN production in myeloid cells (2), resulting in a phenotype with stronger IFN elements, classifying it as SLE.

In addition to being identified as a risk gene in SLE GWAS studies (24), the aggregation of rare variants in *IKBKE* has been observed in individuals with SLE and RA (37, 38), underscoring the significance of this gene in immune-inflammatory conditions. IKKϵ is constitutively expressed in T cells, although its expression is mainly regulated by NFκB in other cell types (4, 5). First, transfection into HEK293 reporter cells demonstrated that the variant in this case attenuates the activation of NFκB and type I IFN signaling (**Figures 3B, C**). A comprehensive evaluation based on RNA-seq data of immune cell subsets revealed downregulation of type I IFN genes in neutrophils (**Figures 4B, C**, **Supplementary Table S2C**). Splice variants of *IKBKE* lacking exon 20 or 21 have been reported to have the potential to inhibit the activation of NFκB and/or IRF3 in a dominant-negative manner due to their inability to bind to adapter proteins NAP1, TANK, and SINTBAD (9). The structure of IKKϵ remains somewhat elusive but based on amino acid sequencing, it is thought to closely resemble IKKβ and is assumed to comprise a kinase domain, a ubiquitin-like domain (ULD), and a long α-helix structure dimerization domain (SDD) (4, 5, 39, 40). The *IKBKE* C626Y variant is located in exon 19 at the 1877^th^ base pair (626^th^ amino acid), likely within the SDD (**Supplementary Figure S2**). The structural change here may interfere with IKKϵ homodimer formation, binding to TBK1, and interaction with adaptor proteins, potentially leading to reduced activity of IKKϵ, thereby resulting in decreased signaling of NFκB and type I IFN.

We consider the lack of reproducibility in confirming whether the *IKBKE* variant is the cause of the pathology in this case as the main limitation of our study. However, the identified *IKBKE* variant is a novel germline variant not previously reported and its functionality has been confirmed through *in vitro* experiments, suggesting a potential role in the pathology. Accumulation of similar cases is necessary to determine if the *IKBKE* variant is pathogenic. Another limitation is that changes have been observed in immune cells other than T cells, such as NK cells, B cells, monocytes, and neutrophils, raising the possibility that these changes may be more significant in the pathology than those in T cells. While our examination of immune cell subset ratios and RNA-seq is highly comprehensive, a larger number of DEGs in a particular subset does not necessarily mean it has a greater contribution to the disease and there is a possibility of larger changes occurring in immune cells or tissues not analyzed. Specifically, in cell types other than T cells, the expression of *IKBKE* is primarily regulated by NFκB, and as shown in **Figure 3B**, this variant significantly reduced NFκB and type I IFN, suggesting the potential influence of other cells on the pathology in this case. Additionally, while the association between the variant and the observed immune cell abnormalities and disease phenotype is inferred from the literature, future studies using genetically modified mice could directly demonstrate the causal relationship, thereby enhancing our understanding of the disease mechanisms.

In summary, the *IKBKE* variant can reduce the activity of IKKϵ, potentially leading to dysregulation of various immune mechanisms and induction of inflammation. Targeting functional sites of *IKBKE* or its related molecules could be beneficial not only for patients with similar inflammatory pathology as in this case, but also for those with SLE or RA.

In conclusion, we presented a patient with polyarthritis and fevers and identified a novel heterozygous c.1877G>A, p(Cys626Tyr) variant in *IKBKE*. Loss-of-function in *IKBKE* could contribute to the development of inflammatory pathology by T cell activation. Therefore, *IKBKE* represents a potential target for immunotherapy.

## Data Availability

The datasets presented in this study can be found in online repositories. The names of the repository/repositories and accession number(s) can be found in the article/**Supplementary Material**.
