# Supplementary material for: A novel functional IKBKE variant activating NFAT in a patient with polyarthritis and a remittent fever
Source: Front Immunol. 2024 Oct 25;15:1475179. doi: 10.3389/fimmu.2024.1475179 (PMC11544129; doi:10.3389/fimmu.2024.1475179)
Supplement: Supplementary file 1 [file DataSheet1.docx]

**Supplementary methods**

**Functional analysis experiment of *IKBKE* variants transfected into Jurkat reporter cells.**

Exon sequences containing the rare variant of *IKBKE* or the wild-type were cloned into pcDNA3.1+ N-eGFP plasmids (Gene Script). Additionally, Jurkat-Lucia^TM^ NFAT cells (InvivoGen, San Diego, CA, USA) were prepared. These cells produce both NFAT and luciferase, and when mixed with QUANTI-Luc^TM^ (InvivoGen, San Diego, CA, USA), a substrate for the luciferase reaction, a light signal is generated by the luciferase reaction. This signal can be quantitatively measured using a luminometer to assess the produced NFAT.

Cells were first seeded at a density of 2×10^5^ cells/ml in a 6-well plate and cultured in IMDM supplemented with 10% heat-inactivated fetal bovine serum (FBS), 4-(2-hydroxyethyl)-1-piperazineethanesulfonic acid (HEPES), penicillin/streptomycin and L-Glutamine at 37°C. After 24 hours, one of the following plasmids was transfected into the reporter cells: wild-type *IKBKE*, *IKBKE* variant, or mock control. Specifically, plasmid (2.5 µg), TransIT-Jurkat Reagent as the transfection agent (7.5 µl, takara-bio), and Opti-MEM (225 µl) were thoroughly mixed and stored at room temperature for 30 minutes. Afterward, the mixture was added to each well. The culture was then continued at 37°C.

GFP-positive cells were sorted by flow cytometry using MoFlo XDP sorter (Beckman Coulter) 48 hours after transfection. The gating strategy is shown in Fig. S3. A 24-well plate was pre-warmed with 1000 µl/well at 37℃, and GFP-positive cells were seeded at a density of 35,000 cells/well.

CD3/28 stimulation groups were also prepared. Specifically, 24 hours before sorting, anti-human CD3 antibody (Invitrogen, clone OKT3, 0.5 μg/μL) and anti-human CD28 antibody (BD, clone CD28.2, 0.5 μg/μL) were mixed and diluted with PBS to a final concentration of 5 μg/mL each. Then, 300 μL of the CD3/28 antibody mixture was applied to each well of a 24-well flat plate and incubated at 4°C overnight. The CD3/28 antibody solution was aspirated and discarded, and the wells were washed once with PBS (600 μL/well). Subsequently, the cells were seeded under the same conditions as the non-CD3/28 stimulation groups.

After 24 hours of incubation at 37°C, reporter assays were performed on the sorted cells. 20μL of culture medium was transferred to the wells of a clear-bottom white polystyrene microplate (Corning, Corning NY) for each well, and then mixed with 20μL of QUANTI-Luc™. Luminescence was measured using the GloMax® Navigator Microplate Luminometer.

**Functional analysis experiment of *IKBKE* variants transfected into HEK293 reporter cells.**

Exon sequences containing the rare variants of *IKBKE* or the wild type were cloned into pcDNA3.1+ N-DYK plasmids (Gene Script). Additionally, we prepared two types of reporter cells: Human Tumor necrosis factor (TNF)-α secreted alkaline phosphatase (SEAP) reporter HEK293 cells (InvivoGen, San Diego, CA, USA), and Human Type I interferons (IFNs) reporter HEK293 cells (InvivoGen). The TNF-α SEAP reporter cells were engineered by fusing the SEAP reporter gene with five NFκB binding sites, enabling the detection of TNFα by monitoring the activation of the NFκB pathway. The type I IFNs reporter cells produce SEAP in the presence of type I IFNs. QUANTI-Blue™ (InvivoGen, San Diego, CA, USA) is a colorimetric enzyme assay developed to determine any alkaline phosphatase activity in a biological sample, such as cell culture supernatant.

We coated 6-well plate with 0.01% Poly-L-Lysin (Sigma-Aldrich) at a volume of 0.2 mL/well for 20 minutes at room temperature. After removing excess Poly-L-Lysine, the TNF-α SEAP reporter cells and type I IFNs reporter cells were cultured on the coated plate at a density of 5.0x10^5^ cells/well density for each cell type in DMEM supplemented with 10% FBS, HEPES, penicillin/streptomycin and L-Glutamine at 37°C.

24 hours after the final passage, we transfected one of the plasmids to the reporter cells: wild-type *IKBKE*, *IKBKE* with the variant or mock. Specifically, a mixture of plasmid (3.4 µg), FuGENE HD (10.1µl, Promega) as the transfection agent, and Opit-MEM (225 µl) (Thermo Fisher Scientific) was prepared and kept at room temperature for 10 minutes before being added to each well.

After incubating for 48 hours at 37°C, the expression of SEAP in the supernatant of each transfected cell line (20 µl/well) was measured at 620mm using a spectrophotometer, following the manufacturer's instructions and using the QUANTI-Blue.

**Peripheral blood mononuclear cells (PBMC) collection and RNA purification**

Whole blood in an amount of 30 mL of the patient was mixed with 1 mL of heparin and diluted with an equivalent amount of wash buffer (phosphate-buffered saline + 2% fetal calf serum [BioWest] + 1 mM EDTA [ethylenediaminetetraacetic acid]), and the peripheral blood mononuclear cell (PBMC) layer was recovered by density gradient centrifugation (1000 g for 10 minutes at 20 ℃) using the Ficoll-Plaque PLUS (GE Healthcare). Following hemolysis treatment with an ammonium chloride potassium solution (150 mL NH_4_Cl + 10 mM KHCO_3_ + 0.1 mM Na_2_EDTA), the resulting product was washed using a wash buffer. Following non-specific binding inhibition of Fcγ receptors by means of a human Fc receptor binding inhibitor (eBioscience), the resulting product was stained using various fluorescently labelled antibodies. After staining, the resulting product was suspended in a basic sort buffer (Hank’s balanced salt solution + 2% fetal calf serum + 1 mM EDTA + 25 mM 4-(2-hydroxyethyl)-1-piperazineethanesulfonic acid), and flow cytometry was used for analysis and cell sorting.

**Sorting of immunocompetent cell subsets by flow cytometry**

We sorted 26 cell subsets using the FACS Aria^TM^ Fusion flow cytometer: Naïve CD4 T cells (Naïve CD4), Memory CD4 T cells (Mem CD4), T helper 1 cells (Th1), T helper 2 cells (Th2), T helper 17 cells (Th17), T follicular helper cells (Tfh), Fraction I naïve regulatory T cells (Fr. I nTreg), Fraction II effector regulatory T cells (Fr. II eTreg), Fraction III non-regulatory T cells (Fr. III T), Naïve CD8 T cells (Naïve CD8), Central memory CD8 T cells (CM CD8), Effector memory CD8 T cells (EM CD8), CD8+ T effector memory CD45RA+ cells (TEMRA CD8), Natural killer cells (NK), Naïve B cells (Naïve B), Unswitched memory B cells (USM B), Switched memory B cells (SM B), Double negative B cells (DN B), Plasmablasts (Plasmablast), Classical monocytes (CL Mono), CD16 positive monocytes (CD16p Mono), Intermediate monocytes (Int Mono), Non-classical monocytes (NC Mono), Myeloid dendritic cells (mDC), and Plasmacytoid dendritic cells (pDC). Each subset was sorted with the upper limit being 5,000 cells, and then stored in a deep freezer at -80℃. Furthermore, the fluorescently labeled antibodies that were used in PBMC staining for each dataset are shown in Table S3.

**Collection of peripheral blood neutrophils**

Samples of 3 mL of whole blood were collected using collection tubes containing EDTA-2K. The samples were subjects to magnetic separation using “MACSexpress Neutrophil Isolation Kit, human” kits (Miltenyi Biotec), and the erythrocytes were removed using “MACSexpress Erythrocyte Depletion Kit, human” kits (Miltenyi Biotec). From each sample, around 2×10^6^ cells were then obtained, and lysed using TRIZOL® LS reagent (Invitrogen), and the resulting product was stored in a deep freezer at -80℃.

**cDNA library preparation and RNA-sequencing (RNA-seq)**

Each recovered cell subset sample was subjected to RNA purification using MagMAXTM96 Total RNA Isolation Kit (Thermo Fisher Scientific).

Sequence libraries were prepared using the SMART-seq V4 Ultra Low Input RNA Kit for Sequencing (Clontech Laboratories, Inc.), the Nextera XT DNA Library Prep Kit (Illumina, Inc.), or the Nextera XT Index Kit V2 Set A-D (Illumina, Inc.). The HiSeq 2500 (Illumina, Inc.) system was used to obtain 100 bp paired-end base sequences. The bcl files were demultiplexed and converted into FASTQ files using bcl2fastq2 conversion software v2.17.

**FASTQ file quality control and mapping and counting**

The adapter sequences were removed using Cutadapt v1.14 [1], and FASTX Toolkit v0.0.14 was used to remove bases with a Phred quality score below 20 from the 3’-terminal (http://hannonlab.cshl.edu/fastx_toolkit/), and leads with a base length of less than 50 base sequences were removed. Leads having 20% or more bases with a Phred quality score below 20 were also removed. The USCS human genome 38 (hg38) was used as the reference sequence, mapping was performed using STAR v2.5.3a [2], and counting was performed using HTSeq v0.9.1 [3]. Samples with a uniquely mapped rate below 80% or fewer than 5,000,000 uniquely mapped reads were excluded from subsequent analyses.

**RNA-seq sample differentially expressed gene set analysis.**

A differentially expressed genes (DEGs) analysis of our patient, compared to the HC population in our ImmuNexUT publicly available data [4], who were performed RNA-seq using the same procedure as the patient, was performed for each subset using edgeR v3.24.3, and the log fold change of each gene was calculated [5]. Because we did not have replicates in the patient’s samples, we hypothesized these samples' biological coefficient of variation value were standard (0.4), and tested whether some of the genes were differentially expressed by exact tests with the threshold of false discovery rate 0.05. Additionally, we performed pathway enrichment analysis of DEGs [6].

**Supplementary Figure 1. Filtering strategies for candidate causal single nucleotide variants in the patient.**

From all the variants detected in the patients, we extracted variants that could explain the patients' symptoms. After restricting the variants to those that affect the protein produced, we narrowed the list based on frequency information and registered pathogenicity assessments using ClinVar and The Human Gene Mutation Database.

**Supplementary Figure 2. The structure of IKKε with the wildtype and C626Y variant as inferred by G23D.**

Using G23D [7], an online tool for mapping and visualization of genomic variants on 3D protein structures, we displayed the structure of IKKε with the wildtype and C626Y variant. The red arrow indicates the variant site.

**Supplementary Figure 3. Gating strategy for assessing transfection efficacy.**

Representative flow cytometry plots illustrating gating strategy for assessing transfection efficacy of viable cells. Dead cells were excluded using DAPI, and the transfection efficiency was evaluated based on GFP expression.

**Supplemental Tables S1-3**

**Supplementary Table 1. D****efinitions of the peripheral blood immune cell subsets.**

| **Subset name** | **Abbreviation** | **Definition** |
| --- | --- | --- |
| **CD4 T cells** | **CD4** |  |
| Naïve CD4 T cells | Naïve CD4 | CD3+/CD4+CD8-/CCR7+CD45RA+ |
| Memory CD4 T cells | Mem CD4 | CD3+/CD4+CD8-/non-naive CD4+/CD25- |
| T helper 1 cells | Th1 | CD3+/CD4+CD8-/non-naive CD4+/CD25-/CXCR5-CCR6-/CXCR3+CCR4- |
| T helper 2 cells | Th2 | CD3+/CD4+CD8-/non-naive CD4+/CD25-/CXCR5-CCR6-/CXCR3-CCR4+ |
| T helper 17 cells | Th17 | CD3+/CD4+CD8-/non-naive CD4+/CD25-/CXCR5-CCR6+/CXCR3- |
| T follicular helper cells | Tfh | CD3+/CD4+CD8-/non-naive CD4+/CD25-/CXCR5+ |
| Fraction II effector regulatory T cells | Fr. II eTreg | CD3+/CD4+CD8-/CD25++CD45RA- |
| Fraction I naïve regulatory T cells | Fr. I nTreg | CD3+/CD4+CD8-/CD25+CD45RA+ |
| Fraction III non-regulatory T cells | Fr. III T | CD3+/CD4+CD8-/CD25+CD45RA- |
| **CD8 T cells** | **CD8** |  |
| Naïve CD8 T cells | Naïve CD8 | CD3+CD19-/CD4-CD8+/CD45RA+CCR7+ |
| CD8+ T effector memory CD45RA+ cells | TEMRA CD8 | CD3+CD19-/CD4-CD8+/CD45RA+CCR7- |
| Effector Memory CD8 T cells | EM CD8 | CD3+CD19-/CD4-CD8+/CD45RA-CCR7- |
| Central Memory CD8 T cells | CM CD8 | CD3+CD19-/CD4-CD8+/CD45RA-CCR7+ |
| **Natural Killer cells** | **NK** | CD3-CD19-/CD14-/CD56+ |
| **B cells** | **B** |  |
| Naïve B cells | Naïve B | CD3-CD19+/IgD+CD27- |
| Unswitched memory B cells | USM B | CD3-CD19+/IgD+CD27+ |
| Switched memory B cells | SM B | CD3-CD19+/IgD-CD27+/CD38- |
| Double Negative B cells | DN B | CD3-CD19+/IgD-CD27- |
| Plasmablasts | Plasmablast | CD3-CD19+/IgD-CD27++/CD38+ |
| **Monocytes** | **Mono** |  |
| Classical monocytes | CL Mono | CD3-CD19-/HLA-DR+/CD56-/CD14+CD16- |
| CD16 positive monocytes | CD16p Mono | CD3-CD19-/HLA-DR+/CD56-/CD14+CD16+ |
| Intermediate monocytes | Int Mono | CD3-CD19-/HLA-DR+/CD56-/CD14++CD16+ |
| Non-classical monocytes | NC Mono | CD3-CD19-/HLA-DR+/CD56-/CD14dimCD16+ |
| **Dendric cells** | **DC** |  |
| Myeloid dendritic cells | mDC | CD3-CD19-/HLA-DR+/CD56-/CD14-CD16-/CD11c+CD123- |
| Plasmacytoid dendritic cells | pDC | CD3-CD19-/HLA-DR+/CD56-/CD14-CD16-/CD11c-CD123+ |
| **Neutrophils** | **Neu** | Immune-magnetically sorting with the "MACSxpress Neutrophil isolation Kit, human" |

**Supplementary Table 2A. Differential gene expression analysis of TEMRA CD8^+^ T cells between our patient and healthy controls.**

| **id** | **symbol** | **logFC** | **logCPM** | **PValue** |
| --- | --- | --- | --- | --- |
| ENSG00000164112.12 | TMEM155 | 5.320106111 | -0.8707543 | 2.53516E-31 |
| ENSG00000081189.15 | MEF2C | 4.619912859 | -0.065682284 | 1.94647E-29 |
| ENSG00000232208.2 | AL139415.1 | 3.980685183 | 0.844010307 | 1.60403E-23 |
| ENSG00000257924.1 | LINC02416 | 4.657242775 | -0.927854559 | 2.22526E-20 |
| ENSG00000146530.11 | VWDE | 4.706901998 | -1.054102322 | 2.89527E-19 |
| ENSG00000171451.13 | DSEL | 3.478036196 | 1.205105766 | 3.80679E-17 |
| ENSG00000171517.5 | LPAR3 | 3.852082345 | -0.220167234 | 3.01221E-16 |
| ENSG00000169071.14 | ROR2 | 3.70046012 | 0.022345598 | 8.66405E-16 |
| ENSG00000180611.6 | MB21D2 | 3.840708664 | -0.380588336 | 2.39894E-15 |
| ENSG00000100292.16 | HMOX1 | 3.526656603 | 0.362043279 | 2.48528E-15 |
| ENSG00000182511.11 | FES | 3.400033208 | 0.838173421 | 3.83049E-15 |
| ENSG00000204381.11 | LAYN | 4.015786482 | -0.708835431 | 6.44933E-15 |
| ENSG00000273445.1 | AC133644.2 | 3.798866639 | -0.400969087 | 7.96027E-15 |
| ENSG00000275791.1 | TRBV10-3 | 3.079720098 | 2.877873759 | 2.11167E-14 |
| ENSG00000101230.5 | ISM1 | 4.325805238 | -1.165423444 | 8.83977E-14 |
| ENSG00000211828.1 | TRDJ3 | 3.317866086 | 0.47441227 | 2.38354E-13 |
| ENSG00000134545.13 | KLRC1 | 2.930987951 | 4.879349442 | 2.70863E-13 |
| ENSG00000100302.6 | RASD2 | 3.886577371 | -0.783531063 | 4.44152E-13 |
| ENSG00000135077.8 | HAVCR2 | 2.894189247 | 3.991631457 | 8.2593E-13 |
| ENSG00000183087.14 | GAS6 | 3.489577082 | -0.217070282 | 1.6207E-12 |
| ENSG00000211803.2 | TRAV23DV6 | 2.961240613 | 1.513825966 | 8.31717E-12 |
| ENSG00000182809.10 | CRIP2 | 2.833803132 | 3.043393204 | 8.56409E-12 |
| ENSG00000254275.6 | LINC00824 | 3.801658106 | -0.888119876 | 2.08818E-11 |
| ENSG00000012779.10 | ALOX5 | 3.438831332 | -0.483331778 | 9.1689E-11 |
| ENSG00000129521.13 | EGLN3 | 3.198060217 | 0.019751016 | 1.20693E-10 |
| ENSG00000157570.11 | TSPAN18 | 2.947233785 | 0.770980609 | 1.94217E-10 |
| ENSG00000100346.17 | CACNA1I | 3.466805863 | -0.655045585 | 2.36887E-10 |
| ENSG00000113249.12 | HAVCR1 | 3.090254019 | 0.046513218 | 6.56575E-10 |
| ENSG00000145850.8 | TIMD4 | 3.099423274 | -0.069264013 | 1.0425E-09 |
| ENSG00000184451.5 | CCR10 | 3.069254274 | -0.050039795 | 1.5907E-09 |
| ENSG00000127329.15 | PTPRB | 3.484472634 | -0.805733917 | 2.41384E-09 |
| ENSG00000277586.2 | NEFL | 4.88078941 | -1.735888612 | 2.88292E-09 |
| ENSG00000177990.11 | DPY19L2 | 2.849055011 | 0.540650112 | 2.91487E-09 |
| ENSG00000170074.19 | FAM153A | 3.137440399 | -0.240824484 | 3.0397E-09 |
| ENSG00000128040.10 | SPINK2 | 3.218628392 | -0.450478195 | 3.1327E-09 |
| ENSG00000158747.13 | NBL1 | 2.967806354 | 0.155309038 | 3.33705E-09 |
| ENSG00000211777.2 | TRAV3 | 2.498377618 | 3.430645764 | 6.01618E-09 |
| ENSG00000146233.7 | CYP39A1 | 5.503118778 | -1.916305981 | 6.251E-09 |
| ENSG00000140511.11 | HAPLN3 | 2.492379494 | 3.078258434 | 8.97146E-09 |
| ENSG00000173585.15 | CCR9 | 3.526159971 | -1.017331895 | 1.19232E-08 |
| ENSG00000142611.16 | PRDM16 | 3.354588349 | -0.811404038 | 1.32373E-08 |
| ENSG00000070159.13 | PTPN3 | 4.640127715 | -1.698737071 | 1.45292E-08 |
| ENSG00000136634.5 | IL10 | 4.640029377 | -1.698701236 | 1.45292E-08 |
| ENSG00000256590.2 | TRDV3 | 2.59333829 | 1.48701024 | 1.46903E-08 |
| ENSG00000159128.14 | IFNGR2 | 2.695687061 | 0.661478373 | 2.5416E-08 |
| ENSG00000138185.19 | ENTPD1 | 2.554924519 | 1.520337946 | 2.64132E-08 |
| ENSG00000273749.4 | CYFIP1 | 2.495721521 | 1.96742658 | 2.91178E-08 |
| ENSG00000180549.7 | FUT7 | 2.433321724 | 2.808511163 | 3.06203E-08 |
| ENSG00000131686.14 | CA6 | 2.67249883 | 0.743121211 | 3.08577E-08 |
| ENSG00000163421.8 | PROK2 | -9.713923791 | 3.747561355 | 3.29839E-08 |
| ENSG00000114948.12 | ADAM23 | 4.317477388 | -1.599906736 | 3.6573E-08 |
| ENSG00000254750.1 | CASP1P2 | 3.64134535 | -1.235324636 | 3.93198E-08 |
| ENSG00000178445.9 | GLDC | 3.299120708 | -0.826221407 | 4.20695E-08 |
| ENSG00000198574.5 | SH2D1B | 2.360380512 | 4.815245782 | 4.47035E-08 |
| ENSG00000258116.1 | AC008083.2 | 2.962283993 | -0.257903267 | 4.64601E-08 |
| ENSG00000036448.9 | MYOM2 | -5.50448963 | 5.687819842 | 4.85168E-08 |
| ENSG00000154639.18 | CXADR | 3.332141899 | -0.926682044 | 5.71018E-08 |
| ENSG00000105974.11 | CAV1 | 3.550896769 | -1.200230914 | 6.01015E-08 |
| ENSG00000124491.15 | F13A1 | 3.017234972 | -0.469309193 | 6.2506E-08 |
| ENSG00000226757.2 | PP12613 | 5.060577524 | -1.853279465 | 6.94887E-08 |
| ENSG00000283125.1 | AC022726.2 | 3.633448302 | -1.277658192 | 6.99113E-08 |
| ENSG00000168209.4 | DDIT4 | 2.309376175 | 5.893813236 | 8.29766E-08 |
| ENSG00000061337.15 | LZTS1 | 3.755273014 | -1.411593137 | 8.64073E-08 |
| ENSG00000229056.2 | AC020571.1 | 2.9522653 | -0.356364426 | 8.64604E-08 |
| ENSG00000109906.13 | ZBTB16 | 2.443597972 | 1.667798185 | 1.09282E-07 |
| ENSG00000198478.7 | SH3BGRL2 | 2.493841089 | 1.190000516 | 1.29132E-07 |
| ENSG00000261572.1 | AC097639.1 | 4.953895672 | -1.843127322 | 1.35484E-07 |
| ENSG00000121807.5 | CCR2 | 2.319606328 | 3.402342337 | 1.37516E-07 |
| ENSG00000233916.1 | ZDHHC20P1 | 2.933359693 | -0.418883419 | 1.48219E-07 |
| ENSG00000179934.6 | CCR8 | 2.809295596 | -0.024124698 | 1.52036E-07 |
| ENSG00000227051.6 | C14orf132 | 4.094002701 | -1.548942071 | 1.56988E-07 |
| ENSG00000134184.12 | GSTM1 | -9.318229753 | 3.366184855 | 1.6554E-07 |
| ENSG00000120875.8 | DUSP4 | 3.145279017 | -0.746948966 | 1.73514E-07 |
| ENSG00000140675.12 | SLC5A2 | 3.28712382 | -0.996601304 | 2.29027E-07 |
| ENSG00000260314.2 | MRC1 | 4.024781269 | -1.531814072 | 2.41337E-07 |
| ENSG00000005513.9 | SOX8 | 3.388938023 | -1.135665258 | 2.45397E-07 |
| ENSG00000198829.6 | SUCNR1 | 5.189121474 | -1.927140509 | 2.50193E-07 |
| ENSG00000245008.3 | AP001122.1 | 3.250094492 | -0.979546998 | 3.09752E-07 |
| ENSG00000196436.8 | NPIPB15 | -9.156852342 | 3.202763292 | 3.13758E-07 |
| ENSG00000154764.5 | WNT7A | 3.138065765 | -0.833084612 | 4.06995E-07 |
| ENSG00000196411.9 | EPHB4 | 3.265577968 | -1.033674274 | 4.07605E-07 |
| ENSG00000137731.13 | FXYD2 | 2.378298548 | 1.429133756 | 4.49377E-07 |
| ENSG00000127585.11 | FBXL16 | 2.418323153 | 1.161683728 | 5.02355E-07 |
| ENSG00000160712.12 | IL6R | 2.348766251 | 1.597502424 | 5.32737E-07 |
| ENSG00000198520.10 | C1orf228 | 2.279573707 | 2.376118399 | 5.7818E-07 |
| ENSG00000163736.3 | PPBP | 2.72012417 | -0.129014356 | 6.84741E-07 |
| ENSG00000164530.13 | PI16 | 2.259361972 | 2.409698858 | 6.86151E-07 |
| ENSG00000223930.5 | AC109779.1 | 3.985487873 | -1.567805218 | 6.97629E-07 |
| ENSG00000172005.10 | MAL | 2.276379379 | 2.025559549 | 7.9356E-07 |
| ENSG00000168646.12 | AXIN2 | 2.436194196 | 0.839714667 | 7.93757E-07 |
| ENSG00000184613.10 | NELL2 | 2.161780136 | 6.223697637 | 7.96366E-07 |
| ENSG00000118515.11 | SGK1 | 2.335180388 | 1.43392339 | 8.07575E-07 |
| ENSG00000171772.16 | SYCE1 | 3.398297319 | -1.24075694 | 8.68569E-07 |
| ENSG00000188672.17 | RHCE | 2.648163271 | 0.059396739 | 9.24514E-07 |
| ENSG00000138678.10 | GPAT3 | 2.473705724 | 0.523015945 | 1.00449E-06 |
| ENSG00000265527.1 | MIR5690 | 2.567145713 | 0.151822329 | 1.11896E-06 |
| ENSG00000174175.16 | SELP | 2.621876276 | 0.043235923 | 1.38568E-06 |
| ENSG00000183780.12 | SLC35F3 | 3.224227663 | -1.117318806 | 1.39752E-06 |
| ENSG00000174255.6 | ZNF80 | 2.137455494 | 4.103216736 | 1.53626E-06 |
| ENSG00000242473.1 | KIR2DP1 | 2.344214582 | 1.020217781 | 2.10071E-06 |
| ENSG00000156510.12 | HKDC1 | 2.311315555 | 1.111240102 | 2.19009E-06 |
| ENSG00000260807.6 | AC009041.2 | 2.846357895 | -0.580578207 | 2.43026E-06 |
| ENSG00000259583.2 | AC015712.2 | 4.697065353 | -1.853687096 | 2.51719E-06 |
| ENSG00000204644.9 | ZFP57 | -8.611674395 | 2.667904977 | 2.63428E-06 |
| ENSG00000109321.10 | AREG | 2.637849421 | -0.231872766 | 2.68203E-06 |
| ENSG00000162843.17 | WDR64 | 3.196522884 | -1.16018576 | 2.70389E-06 |
| ENSG00000114812.12 | VIPR1 | 2.208793404 | 1.706831431 | 3.11009E-06 |
| ENSG00000148773.13 | MKI67 | 2.149463059 | 2.261847894 | 3.74567E-06 |
| ENSG00000088280.18 | ASAP3 | 2.990424765 | -0.905988355 | 3.87649E-06 |
| ENSG00000152078.9 | TMEM56 | 4.597939501 | -1.84358744 | 4.2184E-06 |
| ENSG00000163082.9 | SGPP2 | 2.647254781 | -0.36937985 | 4.27306E-06 |
| ENSG00000107104.18 | KANK1 | 2.109260931 | 2.737910628 | 4.41057E-06 |
| ENSG00000159640.15 | ACE | 2.160944385 | 1.880712154 | 4.75155E-06 |
| ENSG00000156535.13 | CD109 | 2.831178926 | -0.667632338 | 4.87176E-06 |
| ENSG00000136997.17 | MYC | 2.038485086 | 5.369111057 | 4.93527E-06 |
| ENSG00000162576.16 | MXRA8 | 3.163562078 | -1.204037856 | 6.00619E-06 |
| ENSG00000248358.2 | AC243972.1 | 4.237483618 | -1.754272917 | 6.34648E-06 |
| ENSG00000170293.8 | CMTM8 | 2.724720963 | -0.50765512 | 6.52736E-06 |
| ENSG00000174136.11 | RGMB | 2.507353405 | -0.024543092 | 6.5708E-06 |
| ENSG00000227242.4 | NBPF13P | 3.608149098 | -1.468396895 | 7.1132E-06 |
| ENSG00000134765.9 | DSC1 | 2.104056827 | 2.162655292 | 7.18811E-06 |
| ENSG00000100600.14 | LGMN | 2.27726618 | 0.752825151 | 8.09045E-06 |
| ENSG00000128591.15 | FLNC | 4.730566048 | -1.895283002 | 8.24352E-06 |
| ENSG00000177519.3 | RPRM | 4.179364943 | -1.74570515 | 8.51271E-06 |
| ENSG00000259219.1 | AC084855.2 | 4.180549798 | -1.746045082 | 8.51271E-06 |
| ENSG00000066468.22 | FGFR2 | -8.301104191 | 2.378371114 | 8.65607E-06 |
| ENSG00000171408.13 | PDE7B | 3.051099108 | -1.099935688 | 8.71492E-06 |
| ENSG00000261804.1 | AC007342.4 | 2.875999081 | -0.848328781 | 8.8106E-06 |
| ENSG00000156299.13 | TIAM1 | 2.09563357 | 2.040981074 | 8.83244E-06 |
| ENSG00000117602.11 | RCAN3 | 1.997016254 | 4.643682543 | 9.0272E-06 |
| ENSG00000091181.19 | IL5RA | -4.900245006 | 3.177282697 | 9.0629E-06 |
| ENSG00000110777.11 | POU2AF1 | 3.68207124 | -1.542537762 | 9.90573E-06 |
| ENSG00000145107.15 | TM4SF19 | -4.565144796 | 3.394756214 | 1.08875E-05 |
| ENSG00000227416.2 | AL929472.1 | 4.787205839 | -1.93805553 | 1.10693E-05 |
| ENSG00000267626.1 | AC002115.1 | 4.787205839 | -1.93805553 | 1.10693E-05 |
| ENSG00000076706.16 | MCAM | 3.136136471 | -1.252725604 | 1.11945E-05 |
| ENSG00000233695.2 | GAS6-AS1 | 2.63314851 | -0.40654041 | 1.20311E-05 |
| ENSG00000161921.14 | CXCL16 | 2.417110374 | 0.078340769 | 1.30942E-05 |
| ENSG00000173530.5 | TNFRSF10D | 2.052169577 | 2.276153979 | 1.3323E-05 |
| ENSG00000073282.12 | TP63 | 3.453570298 | -1.422139889 | 1.65231E-05 |
| ENSG00000065361.14 | ERBB3 | 2.376187222 | 0.147370725 | 1.69062E-05 |
| ENSG00000107281.9 | NPDC1 | 1.984158018 | 2.967238918 | 1.80483E-05 |
| ENSG00000172031.6 | EPHX4 | -5.255405618 | 2.623581643 | 2.11699E-05 |
| ENSG00000255042.3 | AC109635.4 | 4.255057923 | -1.802911616 | 2.12604E-05 |
| ENSG00000110944.8 | IL23A | 2.026061839 | 2.041615322 | 2.21983E-05 |
| ENSG00000164300.16 | SERINC5 | 1.919478324 | 4.462252828 | 2.40942E-05 |
| ENSG00000169994.18 | MYO7B | 4.490259362 | -1.874505344 | 2.85138E-05 |
| ENSG00000248943.1 | AC140125.1 | 4.488919507 | -1.874124241 | 2.85138E-05 |
| ENSG00000275585.2 | AC241377.2 | 4.488784175 | -1.874084742 | 2.85138E-05 |
| ENSG00000227507.2 | LTB | 1.889795458 | 6.165063984 | 2.8982E-05 |
| ENSG00000080854.14 | IGSF9B | 2.148770674 | 0.905970125 | 2.91312E-05 |
| ENSG00000088882.7 | CPXM1 | 3.473270033 | -1.485897112 | 2.92182E-05 |
| ENSG00000136002.18 | ARHGEF4 | 2.045553652 | 1.544824404 | 2.9741E-05 |
| ENSG00000189013.14 | KIR2DL4 | 2.068028147 | 1.298835664 | 3.05313E-05 |
| ENSG00000110077.14 | MS4A6A | 2.679269235 | -0.742366958 | 3.21231E-05 |
| ENSG00000011590.13 | ZBTB32 | 2.147416313 | 0.723210503 | 3.22894E-05 |
| ENSG00000110987.8 | BCL7A | 2.139384405 | 0.757254329 | 3.41093E-05 |
| ENSG00000211825.1 | TRDJ1 | 1.900427083 | 3.73968648 | 3.45112E-05 |
| ENSG00000229769.2 | TRBV10-2 | -4.285260113 | 3.512999023 | 3.45256E-05 |
| ENSG00000249738.9 | AC008691.1 | 3.297972897 | -1.371857636 | 3.4655E-05 |
| ENSG00000089685.14 | BIRC5 | 2.197088065 | 0.46456605 | 3.76659E-05 |
| ENSG00000215788.9 | TNFRSF25 | 1.884217465 | 4.248112969 | 3.7811E-05 |
| ENSG00000163251.3 | FZD5 | 2.907902721 | -1.161076564 | 4.30685E-05 |
| ENSG00000243629.1 | LINC00880 | 2.788109119 | -0.978945447 | 4.32532E-05 |
| ENSG00000101115.12 | SALL4 | 3.252482327 | -1.356527888 | 4.35172E-05 |
| ENSG00000161270.19 | NPHS1 | 4.61698401 | -1.927094821 | 4.35267E-05 |
| ENSG00000227945.1 | AL590006.1 | 2.631977217 | -0.715509632 | 4.54004E-05 |
| ENSG00000185133.13 | INPP5J | 3.809514728 | -1.680542576 | 4.56423E-05 |
| ENSG00000225968.7 | ELFN1 | 3.807498225 | -1.679947579 | 4.56423E-05 |
| ENSG00000169291.9 | SHE | 4.380157555 | -1.863218315 | 4.65866E-05 |
| ENSG00000254851.1 | AP005018.2 | -7.82080719 | 1.920954624 | 4.97617E-05 |
| ENSG00000142192.20 | APP | 2.161318477 | 0.519033575 | 5.38949E-05 |
| ENSG00000048052.21 | HDAC9 | -3.71815321 | 4.083854081 | 5.46093E-05 |
| ENSG00000166145.14 | SPINT1 | 2.161589231 | 0.420736971 | 5.65116E-05 |
| ENSG00000262714.1 | AC007342.5 | 2.647986498 | -0.786407155 | 5.75457E-05 |
| ENSG00000197635.9 | DPP4 | 1.843588845 | 4.392780716 | 5.82734E-05 |
| ENSG00000157456.7 | CCNB2 | 2.253911412 | 0.11936759 | 6.02228E-05 |
| ENSG00000158869.10 | FCER1G | 2.031370097 | 1.082512062 | 6.24863E-05 |
| ENSG00000165272.14 | AQP3 | 1.850112187 | 3.77458215 | 6.32975E-05 |
| ENSG00000226240.1 | LINC00381 | 2.564323386 | -0.676216628 | 6.65457E-05 |
| ENSG00000143365.17 | RORC | 2.130597698 | 0.514927345 | 6.84838E-05 |
| ENSG00000260896.5 | LINC02170 | 3.978050512 | -1.763474637 | 6.90273E-05 |
| ENSG00000164076.16 | CAMKV | 4.283205423 | -1.853675076 | 7.17691E-05 |
| ENSG00000211781.3 | TRAV7 | 4.283115934 | -1.853651012 | 7.17691E-05 |
| ENSG00000101493.10 | ZNF516 | 2.107317295 | 0.692609645 | 7.27133E-05 |
| ENSG00000166002.6 | SMCO4 | 1.93238595 | 1.753563701 | 7.5007E-05 |
| ENSG00000145390.11 | USP53 | 1.816973078 | 4.408759888 | 7.88651E-05 |
| ENSG00000135925.8 | WNT10A | 2.798254343 | -1.114797926 | 8.31303E-05 |
| ENSG00000126353.3 | CCR7 | 1.793383151 | 6.515460094 | 8.46344E-05 |
| ENSG00000178562.17 | CD28 | 1.803713313 | 4.780955009 | 8.63159E-05 |
| ENSG00000231799.2 | PA2G4P6 | 1.887259778 | 2.297357126 | 8.74E-05 |
| ENSG00000180801.13 | ARSJ | 3.920526386 | -1.755013682 | 8.8615E-05 |
| ENSG00000279741.1 | AC007342.8 | 3.630606271 | -1.643549953 | 9.66049E-05 |
| ENSG00000242522.1 | KLHL6-AS1 | 2.592756942 | -0.816078412 | 9.93103E-05 |
| ENSG00000163697.16 | APBB2 | 4.466097236 | -1.916634779 | 0.000106978 |
| ENSG00000248711.1 | THUMPD3P1 | 4.465260117 | -1.916394637 | 0.000106978 |
| ENSG00000174951.10 | FUT1 | 3.394303574 | -1.524773638 | 0.000108181 |
| ENSG00000214189.8 | ZNF788 | 2.459984696 | -0.557517092 | 0.000109238 |
| ENSG00000187260.15 | WDR86 | 2.000738618 | 1.008170078 | 0.000112141 |
| ENSG00000170954.11 | ZNF415 | 2.18220141 | 0.128243746 | 0.000118894 |
| ENSG00000178038.16 | ALS2CL | 2.001851817 | 0.868486718 | 0.00012062 |
| ENSG00000186891.13 | TNFRSF18 | 1.988234265 | 1.046807324 | 0.000123053 |
| ENSG00000270557.1 | AC013731.1 | 2.359514643 | -0.327582614 | 0.000124055 |
| ENSG00000240535.8 | AC034238.1 | 1.813254461 | 2.84751108 | 0.000125461 |
| ENSG00000188266.13 | HYKK | 2.203257228 | -0.022278285 | 0.000129752 |
| ENSG00000237429.1 | BX293535.1 | 2.728835999 | -1.084101274 | 0.000130347 |
| ENSG00000101292.7 | PROKR2 | 2.429992355 | -0.541437786 | 0.000137004 |
| ENSG00000104728.15 | ARHGEF10 | -3.767849621 | 3.317242486 | 0.000143616 |
| ENSG00000006468.13 | ETV1 | 2.371220589 | -0.392019062 | 0.000144635 |
| ENSG00000283499.1 | U6 | 2.367400454 | -0.38911143 | 0.000144635 |
| ENSG00000228113.7 | AC003991.1 | 2.745362208 | -1.162058616 | 0.000151061 |
| ENSG00000211667.3 | IGLV3-12 | -7.500683717 | 1.622041156 | 0.000151366 |
| ENSG00000185710.9 | SMG1P4 | -3.551663976 | 3.568125136 | 0.000160873 |
| ENSG00000178386.12 | ZNF223 | 1.871067307 | 1.769265801 | 0.000161839 |
| ENSG00000258511.1 | LINC02295 | 2.17375702 | -0.004982445 | 0.00016956 |
| ENSG00000127377.8 | CRYGN | 3.748362397 | -1.725957673 | 0.000173265 |
| ENSG00000136854.19 | STXBP1 | 1.847443358 | 1.920166199 | 0.000174323 |
| ENSG00000211804.3 | TRDV1 | 1.728027707 | 5.039740385 | 0.000182829 |
| ENSG00000124507.10 | PACSIN1 | 2.030315863 | 0.513867574 | 0.000184548 |
| ENSG00000213058.3 | AL365357.1 | -7.443881557 | 1.579735367 | 0.000186266 |
| ENSG00000092096.16 | SLC22A17 | 2.124507477 | 0.166325887 | 0.000191566 |
| ENSG00000174885.12 | NLRP6 | 1.810562216 | 2.158050095 | 0.000192227 |
| ENSG00000161835.10 | GRASP | 2.127092918 | 0.123229998 | 0.000196523 |
| ENSG00000156453.13 | PCDH1 | -3.29119891 | 3.946616442 | 0.000205134 |
| ENSG00000124196.5 | GTSF1L | 4.0198679 | -1.823165566 | 0.000205188 |
| ENSG00000071909.18 | MYO3B | -3.5610138 | 3.363093724 | 0.00020966 |
| ENSG00000132970.12 | WASF3 | 4.327777241 | -1.905709824 | 0.000210351 |
| ENSG00000214376.5 | VSTM5 | 4.328495687 | -1.905916354 | 0.000210351 |
| ENSG00000242736.1 | TRBV1 | 2.628871246 | -1.03782182 | 0.000211491 |
| ENSG00000122043.10 | LINC00544 | 3.439013317 | -1.600257757 | 0.000213654 |

**Supplementary Table 2B. Differential gene expression analysis of Natural Killer cells between our patient and healthy controls.**

| **id** | **symbol** | **logFC** | **logCPM** | **PValue** |
| --- | --- | --- | --- | --- |
| ENSG00000158481.12 | CD1C | 7.699272014 | 0.792913183 | 2.1998E-161 |
| ENSG00000179639.10 | FCER1A | 7.124439258 | 1.827757516 | 3.05E-157 |
| ENSG00000110077.14 | MS4A6A | 7.215278003 | 0.151670493 | 1.2541E-115 |
| ENSG00000131401.11 | NAPSB | 6.435730573 | 1.712114872 | 4.4331E-115 |
| ENSG00000139970.16 | RTN1 | 7.115551837 | -0.080982722 | 1.6347E-103 |
| ENSG00000085265.10 | FCN1 | 6.841696767 | 0.045770617 | 5.7349E-97 |
| ENSG00000197629.5 | MPEG1 | 6.332643555 | 0.843107397 | 7.74426E-96 |
| ENSG00000106066.14 | CPVL | 6.011722875 | 1.573059168 | 4.65303E-92 |
| ENSG00000122025.14 | FLT3 | 6.236416634 | 0.63942676 | 4.54427E-88 |
| ENSG00000038427.15 | VCAN | 8.729249556 | -1.13785456 | 6.99385E-88 |
| ENSG00000010610.9 | CD4 | 6.578916825 | 0.011136481 | 4.74755E-86 |
| ENSG00000156886.11 | ITGAD | 5.740145083 | 2.511328756 | 5.32293E-84 |
| ENSG00000163563.7 | MNDA | 5.864471124 | 1.157259748 | 1.54818E-80 |
| ENSG00000114013.15 | CD86 | 6.366403456 | -0.096879302 | 6.21086E-76 |
| ENSG00000132514.13 | CLEC10A | 6.318130249 | -0.022701141 | 6.67661E-76 |
| ENSG00000177575.12 | CD163 | 7.978756112 | -1.15105139 | 1.53452E-74 |
| ENSG00000149418.10 | ST14 | 5.926284297 | 0.424357357 | 1.13986E-71 |
| ENSG00000158473.6 | CD1D | 6.768190358 | -0.646741114 | 9.23125E-71 |
| ENSG00000124491.15 | F13A1 | 6.264388345 | -0.27551818 | 2.20512E-68 |
| ENSG00000135218.17 | CD36 | 6.306747195 | -0.359567264 | 4.10743E-67 |
| ENSG00000116990.10 | MYCL | 6.231637943 | -0.278259774 | 8.92694E-67 |
| ENSG00000134061.5 | CD180 | 6.786891239 | -0.793248938 | 5.03945E-66 |
| ENSG00000160712.12 | IL6R | 6.030422887 | -0.142689107 | 4.05041E-64 |
| ENSG00000128815.19 | WDFY4 | 6.048509763 | -0.177402439 | 2.07849E-63 |
| ENSG00000121316.10 | PLBD1 | 5.644408508 | 0.387911198 | 1.62275E-61 |
| ENSG00000167600.13 | CYP2S1 | 6.715262941 | -0.879819953 | 1.2666E-60 |
| ENSG00000101336.13 | HCK | 5.780030982 | 0.090015301 | 1.54975E-60 |
| ENSG00000178175.11 | ZNF366 | 6.86571998 | -1.013050796 | 5.18261E-59 |
| ENSG00000157445.14 | CACNA2D3 | 6.846926377 | -1.018705791 | 2.04581E-58 |
| ENSG00000166825.13 | ANPEP | 6.090691565 | -0.608565304 | 5.71745E-54 |
| ENSG00000162692.10 | VCAM1 | 5.923822217 | -0.461350602 | 9.61718E-54 |
| ENSG00000166428.12 | PLD4 | 5.425460247 | 0.332837535 | 1.85092E-53 |
| ENSG00000131203.12 | IDO1 | 8.016834782 | -1.495966978 | 7.99565E-53 |
| ENSG00000204472.12 | AIF1 | 5.612532568 | -0.091350374 | 1.36617E-52 |
| ENSG00000163220.10 | S100A9 | 5.401886464 | 0.294873172 | 1.7138E-52 |
| ENSG00000158488.15 | CD1E | 7.538939227 | -1.401241009 | 1.83147E-52 |
| ENSG00000178695.5 | KCTD12 | 6.613782037 | -1.050063327 | 3.23931E-52 |
| ENSG00000138678.10 | GPAT3 | 5.648891188 | -0.210322302 | 4.48367E-51 |
| ENSG00000012779.10 | ALOX5 | 5.347619003 | 0.077620959 | 8.23826E-48 |
| ENSG00000100079.6 | LGALS2 | 6.936873538 | -1.314680086 | 3.73251E-47 |
| ENSG00000163694.14 | RBM47 | 6.279597519 | -1.004317351 | 7.49968E-47 |
| ENSG00000185215.8 | TNFAIP2 | 5.217533268 | 0.24774027 | 1.0588E-46 |
| ENSG00000116701.14 | NCF2 | 5.045168045 | 0.683375433 | 1.8467E-46 |
| ENSG00000142611.16 | PRDM16 | 5.361663452 | -0.123075353 | 4.77736E-45 |
| ENSG00000087253.12 | LPCAT2 | 5.641211869 | -0.597423818 | 2.81909E-43 |
| ENSG00000117115.12 | PADI2 | 6.454120986 | -1.206198744 | 5.18032E-43 |
| ENSG00000120708.16 | TGFBI | 4.91092034 | 0.667536284 | 9.24472E-43 |
| ENSG00000121552.3 | CSTA | 6.847239081 | -1.396372505 | 5.03682E-42 |
| ENSG00000135898.9 | GPR55 | 5.326513005 | -0.321456862 | 3.13977E-41 |
| ENSG00000111275.12 | ALDH2 | 4.686799505 | 1.309275007 | 4.75108E-41 |
| ENSG00000129993.14 | CBFA2T3 | 5.378742335 | -0.45868857 | 2.34111E-40 |
| ENSG00000196189.12 | SEMA4A | 4.570184038 | 1.780726752 | 3.1801E-40 |
| ENSG00000182578.13 | CSF1R | 4.920217313 | 0.314572272 | 9.29083E-40 |
| ENSG00000119535.17 | CSF3R | 5.013218649 | 0.1133775 | 1.31249E-39 |
| ENSG00000066336.11 | SPI1 | 5.082519983 | -0.048053598 | 1.68019E-39 |
| ENSG00000196562.14 | SULF2 | 4.53866926 | 1.757637879 | 2.87283E-39 |
| ENSG00000153823.18 | PID1 | 7.151070003 | -1.545007689 | 4.5453E-39 |
| ENSG00000187164.19 | SHTN1 | 5.762849434 | -0.943226288 | 4.433E-38 |
| ENSG00000174123.10 | TLR10 | 5.649271801 | -0.855404387 | 5.19741E-38 |
| ENSG00000169116.11 | PARM1 | 6.1058365 | -1.193866274 | 2.01045E-37 |
| ENSG00000196576.14 | PLXNB2 | 4.820777319 | 0.30325924 | 5.37636E-37 |
| ENSG00000160593.18 | JAML | 4.283544374 | 2.78266313 | 1.33789E-35 |
| ENSG00000176788.8 | BASP1 | 4.548622592 | 0.958657338 | 1.55705E-35 |
| ENSG00000164125.15 | FAM198B | 6.606449349 | -1.464580137 | 2.19253E-35 |
| ENSG00000181631.6 | P2RY13 | 5.614026065 | -0.949367163 | 3.10879E-35 |
| ENSG00000095585.16 | BLNK | 5.403590243 | -0.755087001 | 5.61016E-35 |
| ENSG00000188389.10 | PDCD1 | 4.96512623 | -0.21238866 | 9.23116E-35 |
| ENSG00000137462.6 | TLR2 | 5.002893133 | -0.299393034 | 3.4291E-34 |
| ENSG00000064225.12 | ST3GAL6 | 5.856982113 | -1.16012856 | 5.25013E-34 |
| ENSG00000135363.11 | LMO2 | 6.159049971 | -1.31670341 | 5.46633E-34 |
| ENSG00000109436.7 | TBC1D9 | 5.738908462 | -1.099608222 | 1.14923E-33 |
| ENSG00000183023.18 | SLC8A1 | 5.736138287 | -1.113480831 | 1.74602E-33 |
| ENSG00000186407.6 | CD300E | 5.695570823 | -1.102087219 | 2.92314E-33 |
| ENSG00000168913.6 | ENHO | 5.44832917 | -0.908033013 | 3.37977E-33 |
| ENSG00000138119.16 | MYOF | 5.462031943 | -0.956198628 | 4.68506E-32 |
| ENSG00000183087.14 | GAS6 | 5.062540148 | -0.549199096 | 5.4644E-32 |
| ENSG00000175489.9 | LRRC25 | 4.323732035 | 1.174266741 | 1.16859E-31 |
| ENSG00000171631.14 | P2RY6 | 5.231019871 | -0.757419978 | 1.24511E-31 |
| ENSG00000197249.13 | SERPINA1 | 4.833811147 | -0.272728953 | 1.6064E-31 |
| ENSG00000101307.15 | SIRPB1 | 5.039583363 | -0.578352684 | 2.55739E-31 |
| ENSG00000053918.15 | KCNQ1 | 5.570728228 | -1.080448077 | 3.57623E-31 |
| ENSG00000261222.2 | AC064805.1 | 6.191211747 | -1.426201092 | 4.40457E-31 |
| ENSG00000169385.2 | RNASE2 | 6.77968518 | -1.613993604 | 1.76747E-30 |
| ENSG00000074964.16 | ARHGEF10L | 6.808262877 | -1.631613058 | 5.0598E-30 |
| ENSG00000145416.13 | 44621 | 4.397723004 | 0.574488442 | 5.15796E-30 |
| ENSG00000175857.8 | GAPT | 4.801215299 | -0.346726988 | 1.05259E-29 |
| ENSG00000014257.15 | ACPP | 5.612309009 | -1.170661416 | 1.29234E-29 |
| ENSG00000104974.11 | LILRA1 | 5.068949029 | -0.781080851 | 1.05955E-28 |
| ENSG00000204287.13 | HLA-DRA | 3.872816495 | 6.101893241 | 2.66572E-28 |
| ENSG00000105383.14 | CD33 | 4.306424774 | 0.579631665 | 4.69801E-28 |
| ENSG00000119866.20 | BCL11A | 5.290051046 | -1.023384598 | 5.49483E-28 |
| ENSG00000143079.14 | CTTNBP2NL | 5.937745518 | -1.4269622 | 1.03751E-27 |
| ENSG00000186818.12 | LILRB4 | 5.488608611 | -1.168343928 | 1.09288E-27 |
| ENSG00000101439.8 | CST3 | 3.89686584 | 3.917388296 | 1.14414E-27 |
| ENSG00000214787.9 | MS4A4E | 6.846272816 | -1.694099286 | 1.30756E-27 |
| ENSG00000181634.7 | TNFSF15 | 6.533735075 | -1.632090319 | 5.40634E-27 |
| ENSG00000142405.21 | NLRP12 | 6.933121395 | -1.731764439 | 9.63893E-27 |
| ENSG00000100368.13 | CSF2RB | 4.456626011 | -0.068393301 | 2.8211E-26 |
| ENSG00000259674.1 | AC092868.1 | 5.251464854 | -1.078656371 | 3.71756E-26 |
| ENSG00000090382.6 | LYZ | 3.898838691 | 2.197947754 | 4.41873E-26 |
| ENSG00000100095.18 | SEZ6L | 6.6835734 | -1.695219267 | 6.19409E-26 |
| ENSG00000154237.12 | LRRK1 | 5.069989403 | -0.980822873 | 1.35674E-25 |
| ENSG00000100504.16 | PYGL | 5.358304499 | -1.182661369 | 1.5749E-25 |
| ENSG00000163687.13 | DNASE1L3 | 6.484405121 | -1.658146701 | 1.95418E-25 |
| ENSG00000168702.17 | LRP1B | 6.587989594 | -1.685398859 | 2.1786E-25 |
| ENSG00000136205.16 | TNS3 | 5.72962251 | -1.419691481 | 2.78868E-25 |
| ENSG00000091106.18 | NLRC4 | 4.550753866 | -0.44946552 | 2.23492E-24 |
| ENSG00000254415.3 | SIGLEC14 | 5.781193549 | -1.466653584 | 3.93344E-24 |
| ENSG00000159128.14 | IFNGR2 | 4.528473977 | -0.439139003 | 3.98609E-24 |
| ENSG00000000971.15 | CFH | 3.821899485 | 1.926065136 | 4.60394E-24 |
| ENSG00000039068.18 | CDH1 | 4.672856565 | -0.643280466 | 5.20685E-24 |
| ENSG00000140090.17 | SLC24A4 | 4.692542043 | -0.669661567 | 5.85644E-24 |
| ENSG00000102445.18 | RUBCNL | 5.778108139 | -1.465751164 | 7.30309E-24 |
| ENSG00000008394.12 | MGST1 | 6.430020733 | -1.685366678 | 7.99951E-24 |
| ENSG00000276600.4 | RAB7B | 6.666421597 | -1.741361275 | 8.522E-24 |
| ENSG00000113749.7 | HRH2 | 5.426412518 | -1.331233613 | 1.54227E-23 |
| ENSG00000112799.8 | LY86 | 4.07256903 | 0.490691981 | 1.73746E-23 |
| ENSG00000145936.8 | KCNMB1 | 5.966000656 | -1.572316956 | 6.12395E-23 |
| ENSG00000018625.14 | ATP1A2 | 5.822096212 | -1.529166471 | 1.2106E-22 |
| ENSG00000155254.12 | MARVELD1 | 4.777183026 | -0.887317202 | 1.37664E-22 |
| ENSG00000157240.3 | FZD1 | 5.133288492 | -1.210828715 | 3.29229E-22 |
| ENSG00000140749.8 | IGSF6 | 3.816957844 | 1.198857071 | 4.73129E-22 |
| ENSG00000146592.16 | CREB5 | 5.264479 | -1.308901443 | 7.57458E-22 |
| ENSG00000235568.6 | NFAM1 | 4.999693117 | -1.127729148 | 8.1836E-22 |
| ENSG00000260757.1 | AC093520.1 | 5.720479326 | -1.530836585 | 9.4518E-22 |
| ENSG00000204252.13 | HLA-DOA | 3.643673159 | 2.115542275 | 1.96448E-21 |
| ENSG00000178726.6 | THBD | 4.35600453 | -0.435268576 | 3.29407E-21 |
| ENSG00000198053.11 | SIRPA | 5.336968044 | -1.368858841 | 3.95051E-21 |
| ENSG00000261269.1 | AC093278.2 | 6.683671194 | -1.809230783 | 3.95905E-21 |
| ENSG00000284690.1 | AC079325.2 | 5.192163184 | -1.331483637 | 9.35788E-21 |
| ENSG00000161642.17 | ZNF385A | 4.563863812 | -0.816995869 | 1.39812E-20 |
| ENSG00000271605.5 | MILR1 | 4.499569957 | -0.728183654 | 2.01503E-20 |
| ENSG00000159713.10 | TPPP3 | 6.290041382 | -1.74124238 | 2.03749E-20 |
| ENSG00000196735.11 | HLA-DQA1 | 3.499737785 | 3.221119371 | 2.04294E-20 |
| ENSG00000136689.18 | IL1RN | 4.841662593 | -1.138332413 | 1.16791E-19 |
| ENSG00000166145.14 | SPINT1 | 4.391137993 | -0.677321532 | 1.76913E-19 |
| ENSG00000241163.7 | LINC00877 | 5.542347487 | -1.53833954 | 1.9005E-19 |
| ENSG00000174500.12 | GCSAM | 3.526173576 | 1.91968746 | 2.87065E-19 |
| ENSG00000131042.14 | LILRB2 | 4.6881021 | -1.061108325 | 6.54821E-19 |
| ENSG00000170017.12 | ALCAM | 3.571063741 | 1.387871912 | 7.43771E-19 |
| ENSG00000112299.7 | VNN1 | 5.416070496 | -1.513729164 | 9.46077E-19 |
| ENSG00000135373.12 | EHF | 6.463734138 | -1.829553534 | 1.39507E-18 |
| ENSG00000214943.4 | GPR33 | 6.462857343 | -1.829303025 | 1.39507E-18 |
| ENSG00000136630.12 | HLX | 5.329871098 | -1.473551704 | 1.52513E-18 |
| ENSG00000146192.14 | FGD2 | 3.509466799 | 1.651851538 | 1.57912E-18 |
| ENSG00000118508.4 | RAB32 | 3.615941931 | 1.028574077 | 1.79807E-18 |
| ENSG00000105492.15 | SIGLEC6 | 4.408014863 | -0.834791814 | 2.57069E-18 |
| ENSG00000135549.14 | PKIB | 5.16408207 | -1.412936727 | 3.24236E-18 |
| ENSG00000178860.8 | MSC | 3.51839006 | 1.359838226 | 4.39711E-18 |
| ENSG00000114923.16 | SLC4A3 | 4.336961383 | -0.803363385 | 1.02179E-17 |
| ENSG00000113758.13 | DBN1 | 3.348079268 | 2.571953403 | 1.34431E-17 |
| ENSG00000123405.13 | NFE2 | 3.904292734 | -0.103285969 | 1.36282E-17 |
| ENSG00000184371.13 | CSF1 | 3.746161421 | 0.30567805 | 1.59801E-17 |
| ENSG00000136014.11 | USP44 | 3.477191081 | 1.371556864 | 1.65248E-17 |
| ENSG00000152315.4 | KCNK13 | 6.092579657 | -1.779959881 | 1.96056E-17 |
| ENSG00000160883.10 | HK3 | 4.68109239 | -1.176923273 | 2.19842E-17 |
| ENSG00000151490.13 | PTPRO | 5.159873757 | -1.458409632 | 2.43382E-17 |
| ENSG00000235531.9 | MSC-AS1 | 3.605224716 | 0.692607546 | 2.65974E-17 |
| ENSG00000132681.16 | ATP1A4 | 6.199874358 | -1.809320944 | 2.89984E-17 |
| ENSG00000164023.14 | SGMS2 | 4.121667467 | -0.561130077 | 3.33262E-17 |
| ENSG00000198502.5 | HLA-DRB5 | 3.225539168 | 5.178448477 | 5.24843E-17 |
| ENSG00000136826.14 | KLF4 | 4.815041985 | -1.269439771 | 6.41303E-17 |
| ENSG00000135678.11 | CPM | 4.61201347 | -1.178228946 | 9.56892E-17 |
| ENSG00000100302.6 | RASD2 | 4.238683734 | -0.798210104 | 1.38421E-16 |
| ENSG00000134256.12 | CD101 | 3.431757962 | 1.209379742 | 1.71464E-16 |
| ENSG00000269404.6 | SPIB | 4.392135897 | -0.968972121 | 1.72654E-16 |
| ENSG00000060558.3 | GNA15 | 4.424075103 | -1.032041524 | 2.55858E-16 |
| ENSG00000169432.14 | SCN9A | 3.64844192 | 0.247798754 | 3.29993E-16 |
| ENSG00000125538.11 | IL1B | 4.91570947 | -1.398251203 | 5.2596E-16 |
| ENSG00000110079.16 | MS4A4A | 5.339343954 | -1.614875768 | 8.23179E-16 |
| ENSG00000242498.7 | ARPIN | 4.530539981 | -1.176230504 | 9.0187E-16 |
| ENSG00000137672.12 | TRPC6 | 6.354493266 | -1.860016821 | 2.18367E-15 |
| ENSG00000261327.4 | AC134312.5 | 6.354556301 | -1.860034854 | 2.18367E-15 |
| ENSG00000136634.5 | IL10 | 5.483267961 | -1.686208823 | 2.43497E-15 |
| ENSG00000184060.10 | ADAP2 | 3.890071108 | -0.436235705 | 2.70505E-15 |
| ENSG00000121807.5 | CCR2 | 3.168943128 | 2.662390546 | 2.84109E-15 |
| ENSG00000106034.17 | CPED1 | 4.612128939 | -1.257070724 | 3.5795E-15 |
| ENSG00000148680.15 | HTR7 | 5.852576197 | -1.799369611 | 4.82605E-15 |
| ENSG00000158485.10 | CD1B | 6.374030335 | -1.880804061 | 5.60437E-15 |
| ENSG00000159399.9 | HK2 | 3.649937541 | -0.099209562 | 1.00638E-14 |
| ENSG00000162594.15 | IL23R | 3.657832805 | -0.108111401 | 1.00638E-14 |
| ENSG00000263482.3 | ANTXRLP1 | 4.190377816 | -0.935516495 | 1.16729E-14 |
| ENSG00000167642.12 | SPINT2 | 3.20797606 | 1.62914624 | 1.22893E-14 |
| ENSG00000226806.1 | AC011893.1 | 5.756184255 | -1.790463624 | 1.25793E-14 |
| ENSG00000105352.10 | CEACAM4 | 5.068757497 | -1.564142581 | 1.45118E-14 |
| ENSG00000127507.17 | ADGRE2 | 3.52062665 | 0.142691255 | 2.41525E-14 |
| ENSG00000064042.17 | LIMCH1 | 5.376248855 | -1.695161158 | 2.619E-14 |
| ENSG00000127990.15 | SGCE | 3.338911338 | 0.721877888 | 3.30284E-14 |
| ENSG00000186265.9 | BTLA | 4.48916943 | -1.24448557 | 3.70056E-14 |
| ENSG00000282608.1 | ADORA3 | 4.782945788 | -1.444224872 | 4.07017E-14 |
| ENSG00000161921.14 | CXCL16 | 3.751999334 | -0.406331904 | 5.95425E-14 |
| ENSG00000255833.1 | TIFAB | 5.574825147 | -1.770828973 | 6.76904E-14 |
| ENSG00000103313.12 | MEFV | 4.070812778 | -0.910058761 | 7.74962E-14 |
| ENSG00000244482.10 | LILRA6 | 4.723628713 | -1.428832969 | 7.78382E-14 |
| ENSG00000012124.16 | CD22 | 3.760080774 | -0.459920041 | 8.50654E-14 |
| ENSG00000163485.16 | ADORA1 | 5.393237369 | -1.723028095 | 8.62756E-14 |
| ENSG00000150681.9 | RGS18 | 3.203648628 | 1.183178151 | 9.51153E-14 |
| ENSG00000187796.14 | CARD9 | 3.423778797 | 0.26696725 | 1.14913E-13 |
| ENSG00000108405.3 | P2RX1 | 4.180254261 | -1.07316852 | 1.53284E-13 |
| ENSG00000111424.10 | VDR | 4.073249341 | -0.940479457 | 1.53942E-13 |
| ENSG00000082397.17 | EPB41L3 | 4.398936636 | -1.214193905 | 1.55204E-13 |
| ENSG00000100600.14 | LGMN | 3.406621244 | 0.277167273 | 1.69766E-13 |
| ENSG00000187554.11 | TLR5 | 3.500149575 | -0.02440644 | 1.94127E-13 |
| ENSG00000175538.10 | KCNE3 | 4.802292971 | -1.505875703 | 2.20697E-13 |
| ENSG00000197992.6 | CLEC9A | 4.774539222 | -1.499738949 | 3.12167E-13 |
| ENSG00000229391.7 | HLA-DRB6 | 3.013864337 | 2.299951744 | 3.23263E-13 |
| ENSG00000104267.9 | CA2 | 4.494720263 | -1.334436261 | 3.3492E-13 |
| ENSG00000179344.16 | HLA-DQB1 | 2.936249682 | 4.319788861 | 3.45553E-13 |
| ENSG00000166927.12 | MS4A7 | 3.948208706 | -0.853544702 | 4.48813E-13 |
| ENSG00000242193.10 | AL359075.1 | 5.201661815 | -1.695651015 | 4.96161E-13 |
| ENSG00000145391.13 | SETD7 | 3.639773636 | -0.368211556 | 5.79232E-13 |
| ENSG00000239998.5 | LILRA2 | 3.523860527 | -0.244842054 | 9.57491E-13 |
| ENSG00000111729.14 | CLEC4A | 3.118640576 | 1.083647592 | 1.34747E-12 |
| ENSG00000115590.13 | IL1R2 | 5.084793897 | -1.677082081 | 1.38689E-12 |
| ENSG00000216490.3 | IFI30 | 2.87752041 | 4.334147395 | 1.46994E-12 |
| ENSG00000224557.7 | HLA-DPB2 | 4.610733706 | -1.458882879 | 1.55622E-12 |
| ENSG00000239961.2 | LILRA4 | 3.574460037 | -0.378915441 | 2.15944E-12 |
| ENSG00000265527.1 | MIR5690 | 3.603047195 | -0.448251483 | 2.3287E-12 |
| ENSG00000111110.11 | PPM1H | 4.388165212 | -1.332732068 | 2.85758E-12 |
| ENSG00000122870.11 | BICC1 | 4.615174515 | -1.491513726 | 3.95802E-12 |
| ENSG00000165801.9 | ARHGEF40 | 3.545042283 | -0.411584157 | 4.64986E-12 |
| ENSG00000196209.12 | SIRPB2 | 4.444453179 | -1.41418349 | 6.47511E-12 |
| ENSG00000057294.14 | PKP2 | 4.27683409 | -1.297275313 | 9.14125E-12 |
| ENSG00000073737.16 | DHRS9 | 4.830678334 | -1.632549975 | 1.2627E-11 |
| ENSG00000129226.13 | CD68 | 3.224048915 | 0.272879838 | 1.28256E-11 |
| ENSG00000188906.14 | LRRK2 | 4.06207953 | -1.120897847 | 1.31285E-11 |
| ENSG00000135094.10 | SDS | 5.014677265 | -1.694673842 | 1.39207E-11 |
| ENSG00000138449.10 | SLC40A1 | 2.98266731 | 1.135778789 | 2.35097E-11 |
| ENSG00000111817.16 | DSE | 2.909267477 | 1.463071231 | 3.44348E-11 |
| ENSG00000179583.18 | CIITA | 2.738294713 | 3.771267902 | 3.8431E-11 |
| ENSG00000153157.12 | SYCP2L | 4.578596025 | -1.547853007 | 3.86501E-11 |
| ENSG00000079215.13 | SLC1A3 | 4.697168595 | -1.606469788 | 3.92353E-11 |
| ENSG00000171051.8 | FPR1 | 3.67313814 | -0.782511065 | 4.00302E-11 |
| ENSG00000114529.12 | C3orf52 | 3.703563095 | -0.831013613 | 4.34216E-11 |
| ENSG00000161944.16 | ASGR2 | 4.541802733 | -1.539287118 | 5.27506E-11 |
| ENSG00000237541.3 | HLA-DQA2 | 2.820956928 | 2.075070177 | 5.28007E-11 |
| ENSG00000186074.18 | CD300LF | 3.918176667 | -1.062415341 | 5.92268E-11 |
| ENSG00000249437.7 | NAIP | 3.234963475 | 0.015681544 | 6.1358E-11 |
| ENSG00000062282.14 | DGAT2 | 4.323308639 | -1.413633099 | 6.15949E-11 |
| ENSG00000120262.9 | CCDC170 | 4.755946167 | -1.650956786 | 7.54509E-11 |
| ENSG00000143674.10 | MAP3K21 | 3.747133378 | -0.953488496 | 8.20199E-11 |
| ENSG00000116962.14 | NID1 | 5.376619389 | -1.800448152 | 1.04369E-10 |
| ENSG00000213726.5 | RPS2P52 | 3.188748491 | 0.046723861 | 1.22069E-10 |
| ENSG00000144290.16 | SLC4A10 | 2.714317048 | 2.820529872 | 1.57186E-10 |
| ENSG00000120658.13 | ENOX1 | 5.79773874 | -1.912671374 | 1.71E-10 |
| ENSG00000130558.19 | OLFM1 | 5.79773874 | -1.912671374 | 1.71E-10 |
| ENSG00000184292.6 | TACSTD2 | 5.79773874 | -1.912671374 | 1.71E-10 |
| ENSG00000273472.1 | AC096733.2 | 5.79773874 | -1.912671374 | 1.71E-10 |
| ENSG00000105501.12 | SIGLEC5 | 3.832161354 | -1.026362264 | 1.71264E-10 |
| ENSG00000231680.1 | AP003774.3 | 5.69477479 | -1.880993467 | 1.72818E-10 |
| ENSG00000100077.14 | GRK3 | 3.013096078 | 0.529842784 | 1.82283E-10 |
| ENSG00000134769.21 | DTNA | 5.288423648 | -1.790436987 | 2.01976E-10 |
| ENSG00000139572.3 | GPR84 | 5.287306033 | -1.79011389 | 2.01976E-10 |
| ENSG00000163221.8 | S100A12 | 5.289789894 | -1.790822189 | 2.01976E-10 |
| ENSG00000204136.10 | GGTA1P | 4.277802691 | -1.436430204 | 2.15466E-10 |
| ENSG00000104870.12 | FCGRT | 2.682179114 | 3.01265139 | 2.36237E-10 |
| ENSG00000136943.10 | CTSV | 5.069284149 | -1.733642335 | 2.45401E-10 |
| ENSG00000263961.6 | C1orf186 | 4.085275889 | -1.303951686 | 2.58206E-10 |
| ENSG00000163736.3 | PPBP | 3.546464182 | -0.783868112 | 2.93513E-10 |
| ENSG00000103811.15 | CTSH | 2.683535736 | 2.821303979 | 2.95605E-10 |
| ENSG00000125869.9 | LAMP5 | 5.206874131 | -1.780956999 | 3.71354E-10 |
| ENSG00000115919.14 | KYNU | 2.776843452 | 1.528119576 | 4.47636E-10 |
| ENSG00000167641.10 | PPP1R14A | 4.027626781 | -1.28436816 | 4.60332E-10 |
| ENSG00000242265.5 | PEG10 | 3.242396774 | -0.312342298 | 5.55111E-10 |
| ENSG00000162896.5 | PIGR | 5.557861913 | -1.870766494 | 5.59984E-10 |
| ENSG00000204577.11 | LILRB3 | 3.172032176 | -0.124618446 | 6.87262E-10 |
| ENSG00000215559.8 | ANKRD20A11P | -7.15308864 | 5.423469666 | 7.59681E-10 |
| ENSG00000143365.17 | RORC | 3.27476971 | -0.403170837 | 7.66501E-10 |
| ENSG00000184305.14 | CCSER1 | 3.580705058 | -0.913628306 | 8.41353E-10 |
| ENSG00000158477.6 | CD1A | 4.530866221 | -1.643454271 | 9.68181E-10 |
| ENSG00000091436.16 | MAP3K20 | 2.674266769 | 2.077373987 | 9.77181E-10 |
| ENSG00000130147.15 | SH3BP4 | 2.873025361 | 0.764513717 | 1.02285E-09 |
| ENSG00000110987.8 | BCL7A | 3.514087578 | -0.842958556 | 1.12582E-09 |
| ENSG00000104918.7 | RETN | 5.627864023 | -1.9020325 | 1.16651E-09 |
| ENSG00000188676.13 | IDO2 | 5.628062186 | -1.902089341 | 1.16651E-09 |
| ENSG00000092758.15 | COL9A3 | 4.375654916 | -1.572854662 | 1.49134E-09 |
| ENSG00000115828.15 | QPCT | 4.145250258 | -1.436697328 | 1.55271E-09 |
| ENSG00000139132.14 | FGD4 | 3.316874951 | -0.524196833 | 1.84449E-09 |
| ENSG00000161955.16 | TNFSF13 | 2.73749956 | 1.246056941 | 2.25651E-09 |
| ENSG00000223881.1 | AL157402.1 | 4.273880331 | -1.549449845 | 2.59116E-09 |
| ENSG00000136869.13 | TLR4 | 3.296097915 | -0.580419351 | 3.56222E-09 |
| ENSG00000066294.14 | CD84 | 2.502594417 | 4.618089762 | 3.88272E-09 |
| ENSG00000112486.15 | CCR6 | 2.626207366 | 1.734581752 | 4.59196E-09 |
| ENSG00000088882.7 | CPXM1 | 2.692166919 | 1.268196111 | 4.80458E-09 |
| ENSG00000058091.16 | CDK14 | 5.035766478 | -1.789839835 | 5.24466E-09 |
| ENSG00000257539.2 | HSPA8P14 | 5.03622656 | -1.789971188 | 5.24466E-09 |
| ENSG00000280153.1 | AC133065.6 | 2.707146855 | 1.121126738 | 5.36195E-09 |
| ENSG00000116031.8 | CD207 | 5.539881972 | -1.923448635 | 6.251E-09 |
| ENSG00000158258.16 | CLSTN2 | 5.539881972 | -1.923448635 | 6.251E-09 |
| ENSG00000100373.9 | UPK3A | 4.314647547 | -1.598115995 | 6.29266E-09 |
| ENSG00000099337.4 | KCNK6 | 2.531014775 | 2.647922357 | 6.73638E-09 |
| ENSG00000171812.12 | COL8A2 | 3.941619121 | -1.375551028 | 7.94959E-09 |
| ENSG00000164626.8 | KCNK5 | 4.275422889 | -1.589382853 | 8.29598E-09 |
| ENSG00000088827.12 | SIGLEC1 | 3.702766908 | -1.164797841 | 8.77171E-09 |
| ENSG00000172243.17 | CLEC7A | 4.134855783 | -1.514504108 | 9.05502E-09 |
| ENSG00000133246.11 | PRAM1 | 2.619752922 | 1.502776521 | 9.20339E-09 |
| ENSG00000095303.14 | PTGS1 | 2.612746771 | 1.462681847 | 1.21522E-08 |
| ENSG00000168079.16 | SCARA5 | 4.202045444 | -1.572685968 | 1.40581E-08 |
| ENSG00000254048.1 | AC105150.1 | 2.97918338 | -0.086065112 | 1.43322E-08 |
| ENSG00000163492.14 | CCDC141 | 2.468939429 | 2.950317348 | 1.51403E-08 |
| ENSG00000183134.4 | PTGDR2 | 3.836239323 | -1.341410375 | 1.63995E-08 |
| ENSG00000151320.10 | AKAP6 | 4.62093988 | -1.695979614 | 2.07346E-08 |
| ENSG00000184602.5 | SNN | 2.524250179 | 1.834502726 | 2.34096E-08 |
| ENSG00000218823.1 | PAPOLB | 5.213525721 | -1.870814475 | 3.24023E-08 |
| ENSG00000138639.17 | ARHGAP24 | 5.3705163 | -1.912876805 | 3.6651E-08 |
| ENSG00000198959.11 | TGM2 | 4.042264481 | -1.534391713 | 3.69171E-08 |
| ENSG00000168461.12 | RAB31 | 2.425396905 | 2.672454475 | 4.19983E-08 |
| ENSG00000165078.12 | CPA6 | 4.931707158 | -1.810575556 | 4.5116E-08 |
| ENSG00000169403.11 | PTAFR | 2.624842476 | 0.849515672 | 4.52219E-08 |
| ENSG00000073756.11 | PTGS2 | 3.64499646 | -1.229486278 | 4.53908E-08 |
| ENSG00000106537.7 | TSPAN13 | 2.771291004 | 0.267486844 | 5.13892E-08 |
| ENSG00000143546.9 | S100A8 | 3.010828797 | -0.338717637 | 5.55996E-08 |
| ENSG00000134531.9 | EMP1 | 3.87884214 | -1.44425495 | 5.97575E-08 |
| ENSG00000232629.8 | HLA-DQB2 | 3.05536166 | -0.476406786 | 6.2506E-08 |
| ENSG00000112137.17 | PHACTR1 | 2.591755292 | 0.94968046 | 6.93979E-08 |
| ENSG00000255197.5 | AC090559.1 | 3.509327882 | -1.129736252 | 7.45379E-08 |
| ENSG00000211844.1 | TRAJ45 | 3.33875037 | -0.918269396 | 7.75642E-08 |
| ENSG00000160307.9 | S100B | -5.220733691 | 6.010720715 | 9.44E-08 |
| ENSG00000128805.14 | ARHGAP22 | 4.770661072 | -1.790747264 | 1.18727E-07 |
| ENSG00000230113.1 | AC138207.1 | 4.768823039 | -1.79021717 | 1.18727E-07 |
| ENSG00000090530.9 | P3H2 | 3.776238254 | -1.413471752 | 1.23027E-07 |
| ENSG00000229140.8 | CCDC26 | 5.218979769 | -1.902370402 | 1.25362E-07 |
| ENSG00000211846.1 | TRAJ43 | 3.081928717 | -0.697148259 | 1.60207E-07 |
| ENSG00000161647.18 | MPP3 | 4.290825386 | -1.634133841 | 1.69654E-07 |
| ENSG00000163803.12 | PLB1 | 2.695915019 | 0.205491947 | 1.76897E-07 |
| ENSG00000104321.10 | TRPA1 | 4.694261736 | -1.780324095 | 1.82857E-07 |
| ENSG00000114805.16 | PLCH1 | 4.696795852 | -1.78105955 | 1.82857E-07 |
| ENSG00000162711.16 | NLRP3 | 2.416548189 | 1.610655653 | 1.90254E-07 |
| ENSG00000254166.2 | CASC19 | 3.293118016 | -0.942390137 | 1.96091E-07 |
| ENSG00000134184.12 | GSTM1 | -9.263119571 | 3.296557583 | 2.03558E-07 |
| ENSG00000168389.17 | MFSD2A | 3.199854152 | -0.804975223 | 2.04372E-07 |
| ENSG00000100365.14 | NCF4 | 2.606919644 | 0.493567956 | 2.08891E-07 |
| ENSG00000204644.9 | ZFP57 | -9.250492902 | 3.285150648 | 2.14206E-07 |
| ENSG00000172215.5 | CXCR6 | 2.26609926 | 4.193381816 | 2.25575E-07 |
| ENSG00000144893.12 | MED12L | 2.647811471 | 0.357475278 | 2.41789E-07 |
| ENSG00000266733.5 | TBC1D29 | 4.891619542 | -1.840136859 | 2.45306E-07 |
| ENSG00000103184.11 | SEC14L5 | 5.22568566 | -1.93430522 | 2.50193E-07 |
| ENSG00000180767.9 | CHST13 | 5.22568566 | -1.93430522 | 2.50193E-07 |
| ENSG00000187672.12 | ERC2 | 5.22568566 | -1.93430522 | 2.50193E-07 |
| ENSG00000187912.11 | CLEC17A | 4.625184532 | -1.771042839 | 2.73666E-07 |
| ENSG00000109099.13 | PMP22 | 3.748871705 | -1.453948082 | 2.75502E-07 |
| ENSG00000176533.12 | GNG7 | 2.933901006 | -0.444874851 | 2.87976E-07 |
| ENSG00000283288.1 | AC138517.2 | 2.973781469 | -0.550670311 | 2.94711E-07 |
| ENSG00000019169.10 | MARCO | 5.081405687 | -1.891808101 | 3.2672E-07 |
| ENSG00000132669.12 | RIN2 | 5.081170944 | -1.891740461 | 3.2672E-07 |
| ENSG00000235098.8 | ANKRD65 | 5.080910713 | -1.891666021 | 3.2672E-07 |
| ENSG00000155367.15 | PPM1J | 3.536232411 | -1.284345723 | 3.51773E-07 |
| ENSG00000135916.15 | ITM2C | 2.372638865 | 1.712525427 | 3.57651E-07 |
| ENSG00000204482.10 | LST1 | 2.349169376 | 1.837776722 | 4.08719E-07 |
| ENSG00000149256.15 | TENM4 | 4.798816293 | -1.830147421 | 4.18551E-07 |
| ENSG00000185710.9 | SMG1P4 | -9.064650959 | 3.106293389 | 4.50669E-07 |
| ENSG00000163606.10 | CD200R1 | 2.393727119 | 1.312211435 | 5.04008E-07 |
| ENSG00000033627.16 | ATP6V0A1 | 2.569792777 | 0.418835652 | 5.6171E-07 |
| ENSG00000230138.1 | AC119428.2 | 3.37378523 | -1.171505569 | 6.47436E-07 |
| ENSG00000248429.5 | AC098679.1 | 4.955704321 | -1.881297967 | 7.18499E-07 |
| ENSG00000106546.12 | AHR | 2.209705831 | 3.317783926 | 7.62067E-07 |
| ENSG00000260314.2 | MRC1 | 3.726596456 | -1.498217961 | 9.17232E-07 |
| ENSG00000151917.17 | BEND6 | 4.16514843 | -1.6515621 | 1.06522E-06 |
| ENSG00000261040.6 | WFDC21P | 4.162722567 | -1.65084643 | 1.06522E-06 |
| ENSG00000169413.2 | RNASE6 | 2.24001275 | 2.237868192 | 1.16367E-06 |
| ENSG00000196126.11 | HLA-DRB1 | 2.131292222 | 6.850248752 | 1.18108E-06 |
| ENSG00000160505.15 | NLRP4 | 5.056008274 | -1.923565587 | 1.22596E-06 |
| ENSG00000283839.1 | AC096667.1 | 5.056068816 | -1.923582882 | 1.22596E-06 |
| ENSG00000125089.16 | SH3TC1 | 2.145007699 | 4.509694601 | 1.24752E-06 |
| ENSG00000164850.14 | GPER1 | 3.341677166 | -1.210834772 | 1.39561E-06 |
| ENSG00000004809.13 | SLC22A16 | 4.840712136 | -1.871056003 | 1.4046E-06 |
| ENSG00000175471.19 | MCTP1 | 2.288929824 | 1.59218856 | 1.51307E-06 |
| ENSG00000142192.20 | APP | 2.520109977 | 0.240630191 | 1.51506E-06 |
| ENSG00000228314.1 | CYP4F29P | -4.774900295 | 4.586099756 | 1.56827E-06 |
| ENSG00000165795.23 | NDRG2 | 2.144010825 | 3.528371643 | 1.70144E-06 |
| ENSG00000165633.12 | VSTM4 | -5.239955644 | 3.530913822 | 2.05106E-06 |
| ENSG00000115271.10 | GCA | 2.168064516 | 2.653840263 | 2.20207E-06 |
| ENSG00000182022.17 | CHST15 | 3.080995654 | -0.937502825 | 2.38178E-06 |
| ENSG00000181458.10 | TMEM45A | 3.544135129 | -1.44547642 | 2.43381E-06 |
| ENSG00000163520.13 | FBLN2 | 2.280353621 | 1.339166742 | 2.60947E-06 |
| ENSG00000255587.7 | RAB44 | 4.904286095 | -1.912931915 | 3.60457E-06 |
| ENSG00000136848.16 | DAB2IP | 4.34039107 | -1.77145283 | 4.64731E-06 |
| ENSG00000229676.2 | ZNF492 | 3.039625783 | -0.9707022 | 4.75057E-06 |
| ENSG00000174776.10 | WDR49 | 3.419339785 | -1.406530128 | 5.00113E-06 |
| ENSG00000178038.16 | ALS2CL | 3.286100251 | -1.301666132 | 5.06851E-06 |
| ENSG00000074416.13 | MGLL | 2.611268172 | -0.23863334 | 5.75859E-06 |
| ENSG00000185338.4 | SOCS1 | 2.082146153 | 2.885310836 | 5.81801E-06 |
| ENSG00000198752.10 | CDC42BPB | 3.123038406 | -1.121124812 | 6.61596E-06 |
| ENSG00000144218.18 | AFF3 | 2.131380081 | 1.988199326 | 6.69311E-06 |
| ENSG00000278486.1 | YRDCP1 | 4.53992673 | -1.840027125 | 6.70159E-06 |
| ENSG00000196436.8 | NPIPB15 | -4.878635857 | 3.180408256 | 8.51349E-06 |
| ENSG00000187862.11 | TTC24 | 2.101072007 | 2.127390962 | 8.55694E-06 |
| ENSG00000282826.1 | FRG1CP | -4.267826552 | 4.302046822 | 8.64655E-06 |
| ENSG00000241106.6 | HLA-DOB | 2.08788775 | 2.175085437 | 8.79041E-06 |
| ENSG00000104903.4 | LYL1 | 2.823864566 | -0.699044532 | 8.81154E-06 |
| ENSG00000213949.8 | ITGA1 | -8.290140506 | 2.354916238 | 8.90218E-06 |
| ENSG00000128833.12 | MYO5C | 2.578191847 | -0.303672177 | 9.61041E-06 |
| ENSG00000249691.1 | AC026117.1 | 3.124542866 | -1.179800246 | 9.9535E-06 |
| ENSG00000173801.16 | JUP | 2.559514373 | -0.247100952 | 1.02972E-05 |
| ENSG00000279717.1 | AC005336.3 | 3.932593732 | -1.651800846 | 1.06041E-05 |
| ENSG00000136960.12 | ENPP2 | 3.303574652 | -1.368419095 | 1.08381E-05 |
| ENSG00000120162.9 | MOB3B | 3.106253701 | -1.172520366 | 1.09651E-05 |
| ENSG00000104369.4 | JPH1 | 4.82344309 | -1.945242316 | 1.10693E-05 |
| ENSG00000162723.9 | SLAMF9 | 4.82344309 | -1.945242316 | 1.10693E-05 |
| ENSG00000170396.7 | ZNF804A | 4.82344309 | -1.945242316 | 1.10693E-05 |
| ENSG00000175356.12 | SCUBE2 | 4.82344309 | -1.945242316 | 1.10693E-05 |
| ENSG00000186188.10 | FFAR4 | 4.82344309 | -1.945242316 | 1.10693E-05 |
| ENSG00000248747.1 | AC107220.1 | 4.82344309 | -1.945242316 | 1.10693E-05 |
| ENSG00000249661.1 | TNRC18P1 | 4.82344309 | -1.945242316 | 1.10693E-05 |
| ENSG00000260420.2 | LINC02182 | 4.82344309 | -1.945242316 | 1.10693E-05 |
| ENSG00000267325.1 | LINC01415 | 4.82344309 | -1.945242316 | 1.10693E-05 |
| ENSG00000273837.1 | AC018755.4 | 4.155190891 | -1.742627059 | 1.12359E-05 |
| ENSG00000171517.5 | LPAR3 | 2.967635757 | -0.992777401 | 1.14018E-05 |
| ENSG00000163625.15 | WDFY3 | 3.71354509 | -1.547809105 | 1.17104E-05 |
| ENSG00000154556.17 | SORBS2 | -4.73257714 | 3.042671277 | 1.50379E-05 |
| ENSG00000142494.13 | SLC47A1 | 2.089168934 | 1.659601722 | 1.51405E-05 |
| ENSG00000101333.16 | PLCB4 | 3.134064631 | -1.244801609 | 1.53103E-05 |
| ENSG00000153898.12 | MCOLN2 | 1.964848476 | 3.958034553 | 1.53773E-05 |
| ENSG00000146250.6 | PRSS35 | 3.848640559 | -1.633749874 | 1.58272E-05 |
| ENSG00000077942.18 | FBLN1 | 4.641607961 | -1.891866451 | 1.61603E-05 |
| ENSG00000140022.9 | STON2 | 4.042273432 | -1.723321472 | 1.87645E-05 |
| ENSG00000242574.8 | HLA-DMB | 1.938733965 | 4.615429176 | 1.87655E-05 |
| ENSG00000223552.1 | AC098613.1 | 3.302107952 | -1.43025223 | 1.88158E-05 |
| ENSG00000136052.9 | SLC41A2 | 2.564478094 | -0.442885369 | 1.90758E-05 |
| ENSG00000176014.12 | TUBB6 | 2.462226483 | -0.138419742 | 2.05712E-05 |
| ENSG00000141447.17 | OSBPL1A | 3.094409946 | -1.229669776 | 2.06448E-05 |
| ENSG00000204257.14 | HLA-DMA | 1.916106033 | 5.2497942 | 2.33973E-05 |
| ENSG00000138185.19 | ENTPD1 | 2.019604296 | 2.08569382 | 2.38455E-05 |
| ENSG00000109943.8 | CRTAM | 1.909059024 | 5.570466943 | 2.44198E-05 |
| ENSG00000085514.15 | PILRA | 2.240148586 | 0.449644782 | 2.4874E-05 |
| ENSG00000182247.9 | UBE2E2 | 2.222399395 | 0.560277853 | 2.68594E-05 |
| ENSG00000080007.7 | DDX43 | -4.577304589 | 2.886316139 | 2.69989E-05 |
| ENSG00000174171.5 | AC020659.1 | -4.574746116 | 2.890164981 | 2.74475E-05 |
| ENSG00000219392.1 | ZNF602P | 3.021803083 | -1.199822408 | 2.74742E-05 |
| ENSG00000181656.6 | GPR88 | 4.528191673 | -1.882001744 | 2.85138E-05 |
| ENSG00000250687.6 | AC146944.2 | 4.526432854 | -1.88150088 | 2.85138E-05 |
| ENSG00000279275.1 | AP002505.2 | 4.526733145 | -1.881584736 | 2.85138E-05 |
| ENSG00000268849.5 | SIGLEC22P | 2.944085639 | -1.103432937 | 2.92901E-05 |
| ENSG00000105642.15 | KCNN1 | 4.217484038 | -1.800002777 | 2.94525E-05 |
| ENSG00000150540.13 | HNMT | 4.219775352 | -1.800670471 | 2.94525E-05 |
| ENSG00000187116.13 | LILRA5 | 4.219336836 | -1.800546619 | 2.94525E-05 |
| ENSG00000142552.7 | RCN3 | 2.73462157 | -0.758979673 | 3.02709E-05 |
| ENSG00000259641.5 | PCAT29 | 2.714563014 | -0.745123014 | 3.02709E-05 |
| ENSG00000149573.8 | MPZL2 | 2.792855527 | -0.90665956 | 3.43179E-05 |
| ENSG00000123685.8 | BATF3 | 2.517823834 | -0.519522614 | 3.57443E-05 |
| ENSG00000171451.13 | DSEL | 3.127333328 | -1.372579949 | 4.35172E-05 |
| ENSG00000271333.1 | AC019322.2 | 3.12482759 | -1.371790037 | 4.35172E-05 |
| ENSG00000260398.1 | AC068700.1 | 4.653568496 | -1.934365568 | 4.35267E-05 |
| ENSG00000111728.10 | ST8SIA1 | 2.870176303 | -1.069559161 | 4.38022E-05 |
| ENSG00000149451.17 | ADAM33 | 3.043455853 | -1.275288496 | 4.44677E-05 |
| ENSG00000170909.13 | OSCAR | 2.165194925 | 0.578407276 | 4.50081E-05 |
| ENSG00000279970.1 | AC023024.2 | 3.84488887 | -1.687105091 | 4.56423E-05 |
| ENSG00000060982.14 | BCAT1 | 2.731340848 | -0.873587317 | 4.77812E-05 |
| ENSG00000154928.16 | EPHB1 | 3.117131625 | -1.36937803 | 4.86069E-05 |
| ENSG00000146072.6 | TNFRSF21 | 3.017031021 | -1.264597142 | 4.89641E-05 |
| ENSG00000089012.14 | SIRPG | 1.965264757 | 1.845979892 | 4.93826E-05 |
| ENSG00000273749.4 | CYFIP1 | 1.884814582 | 3.211377764 | 5.03908E-05 |
| ENSG00000180340.6 | FZD2 | 2.441306659 | -0.414457407 | 5.04299E-05 |
| ENSG00000155465.18 | SLC7A7 | 2.230674166 | 0.279559802 | 5.08133E-05 |
| ENSG00000196218.12 | RYR1 | 2.091417039 | 0.881902571 | 5.17213E-05 |
| ENSG00000254851.1 | AP005018.2 | -7.790534875 | 1.88733533 | 5.53384E-05 |
| ENSG00000120278.15 | PLEKHG1 | 2.595603683 | -0.621299237 | 5.53388E-05 |
| ENSG00000173482.16 | PTPRM | -3.794159323 | 3.835578245 | 5.53438E-05 |
| ENSG00000256797.1 | KLRF2 | 2.911188068 | -1.154048789 | 5.55771E-05 |
| ENSG00000016391.10 | CHDH | 3.799348446 | -1.678128065 | 5.5684E-05 |
| ENSG00000234424.2 | AL353743.4 | 2.073098514 | 0.777756697 | 6.59774E-05 |
| ENSG00000254294.1 | AC021305.1 | 4.319436832 | -1.860673174 | 7.17691E-05 |
| ENSG00000116574.5 | RHOU | 2.171598788 | 0.400587601 | 7.48627E-05 |
| ENSG00000134369.15 | NAV1 | 2.051205832 | 0.913208345 | 7.65766E-05 |
| ENSG00000186049.8 | KRT73 | -3.894398383 | 3.467375796 | 7.71511E-05 |
| ENSG00000241399.6 | CD302 | 1.946966578 | 1.680249363 | 7.90752E-05 |
| ENSG00000246100.3 | LINC00900 | 2.771976632 | -1.025455805 | 8.52208E-05 |
| ENSG00000116711.9 | PLA2G4A | 2.529780337 | -0.582710364 | 8.63753E-05 |
| ENSG00000157570.11 | TSPAN18 | 2.199697565 | 0.093529184 | 8.97944E-05 |
| ENSG00000166278.14 | C2 | 2.695845844 | -0.921495028 | 9.06011E-05 |
| ENSG00000155974.11 | GRIP1 | 2.345027629 | -0.302675359 | 9.58346E-05 |
| ENSG00000154153.13 | RETREG1 | 1.964117461 | 1.413654048 | 9.84318E-05 |
| ENSG00000160013.8 | PTGIR | 4.501951661 | -1.923683007 | 0.000106978 |
| ENSG00000169397.3 | RNASE3 | 4.502612111 | -1.923871138 | 0.000106978 |
| ENSG00000179593.15 | ALOX15B | 4.501951661 | -1.923683007 | 0.000106978 |
| ENSG00000200252.1 | Y_RNA | 4.501793658 | -1.923637271 | 0.000106978 |
| ENSG00000278226.1 | Metazoa_SRP | 4.502373937 | -1.92380345 | 0.000106978 |
| ENSG00000132694.18 | ARHGEF11 | 2.170123977 | 0.1966887 | 0.000107819 |
| ENSG00000198848.12 | CES1 | 2.664507037 | -0.905641836 | 0.000109269 |
| ENSG00000162722.8 | TRIM58 | 2.150591429 | 0.256682251 | 0.000112482 |
| ENSG00000078900.14 | TP73 | 2.54345298 | -0.710692833 | 0.000123897 |
| ENSG00000134955.11 | SLC37A2 | 2.291542971 | -0.213088235 | 0.000124097 |
| ENSG00000277566.1 | AC089999.3 | 3.589458328 | -1.633590302 | 0.000134939 |
| ENSG00000203616.2 | RHOT1P2 | -7.531188551 | 1.640391383 | 0.000135431 |
| ENSG00000133328.3 | HRASLS2 | -4.086858533 | 2.949210119 | 0.000137634 |
| ENSG00000172031.6 | EPHX4 | -3.876583022 | 3.143760202 | 0.000141166 |
| ENSG00000140479.16 | PCSK6 | 1.866794387 | 1.903198135 | 0.000149895 |
| ENSG00000259162.1 | AL355075.5 | 3.55119801 | -1.624934282 | 0.0001581 |
| ENSG00000169860.6 | P2RY1 | 2.485030646 | -0.619216333 | 0.000162144 |
| ENSG00000177791.11 | MYOZ1 | 3.789081341 | -1.734151318 | 0.000173265 |
| ENSG00000203756.7 | TMEM244 | 2.487834344 | -0.678906578 | 0.000175965 |
| ENSG00000124920.13 | MYRF | 1.788610539 | 2.526116845 | 0.000195789 |
| ENSG00000174944.8 | P2RY14 | 1.946301251 | 1.050335526 | 0.000197353 |
| ENSG00000154146.12 | NRGN | 3.267220013 | -1.485441609 | 0.000202483 |
| ENSG00000104783.11 | KCNN4 | 2.260468424 | -0.354958287 | 0.000204095 |
| ENSG00000117407.16 | ARTN | 4.056649917 | -1.830307732 | 0.000205188 |
| ENSG00000270127.2 | AC027020.2 | -7.408117634 | 1.533652802 | 0.000207173 |
| ENSG00000205037.2 | AC134312.1 | 4.364729315 | -1.913061261 | 0.000210351 |
| ENSG00000174837.14 | ADGRE1 | 2.666566352 | -1.044941863 | 0.000211491 |
| ENSG00000231090.1 | MIR4422HG | 2.502941266 | -0.752456801 | 0.000211742 |
| ENSG00000186854.10 | TRABD2A | 1.90277385 | 1.194799309 | 0.000212291 |
| ENSG00000244649.4 | LINC02086 | 1.935672737 | 0.980546063 | 0.000213124 |
| ENSG00000124731.12 | TREM1 | 3.477548538 | -1.607759948 | 0.000213654 |
| ENSG00000276747.1 | PADI6 | -7.388791674 | 1.515550667 | 0.000220225 |
| ENSG00000166446.14 | CDYL2 | 1.895337327 | 1.228113592 | 0.000221471 |
| ENSG00000149403.12 | GRIK4 | -3.996210952 | 2.32855602 | 0.000229364 |
| ENSG00000153064.11 | BANK1 | 1.928822002 | 0.97551969 | 0.000242605 |
| ENSG00000173530.5 | TNFRSF10D | 2.041126755 | 0.456806607 | 0.000243141 |
| ENSG00000170486.10 | KRT72 | -3.25349479 | 4.060852985 | 0.000252368 |
| ENSG00000183128.7 | CALHM3 | 3.981026223 | -1.821001345 | 0.000274366 |
| ENSG00000233783.7 | AP001442.1 | 3.976693679 | -1.81974055 | 0.000274366 |
| ENSG00000080200.9 | CRYBG3 | 1.8240575 | 1.618599709 | 0.000287489 |
| ENSG00000196420.7 | S100A5 | 2.604031054 | -1.015568966 | 0.000291765 |
| ENSG00000162882.14 | HAAO | 2.84540872 | -1.34799021 | 0.00030977 |
| ENSG00000072110.13 | ACTN1 | 1.695528903 | 3.682038325 | 0.000310871 |
| ENSG00000148655.14 | LRMDA | 2.170717924 | -0.184192986 | 0.000319497 |
| ENSG00000211445.11 | GPX3 | 2.518403914 | -0.900068674 | 0.00033531 |
| ENSG00000074527.11 | NTN4 | -4.446916046 | 1.883193122 | 0.000343868 |
| ENSG00000224011.1 | AC110602.1 | 3.903898299 | -1.810159621 | 0.000358734 |
| ENSG00000232891.1 | AL450344.3 | 4.238833668 | -1.902341929 | 0.000361932 |
| ENSG00000243273.1 | AC020636.1 | 4.239715973 | -1.902595941 | 0.000361932 |
| ENSG00000173546.7 | CSPG4 | 2.558961995 | -0.993550503 | 0.00037254 |
| ENSG00000102780.16 | DGKH | 1.773243713 | 1.585692183 | 0.000426517 |
| ENSG00000105289.14 | TJP3 | -3.778848338 | 2.663113376 | 0.000427189 |
| ENSG00000141574.7 | SECTM1 | 2.131971716 | -0.212421318 | 0.000430125 |
| ENSG00000225000.1 | AC010082.1 | 3.541672093 | -1.687359588 | 0.000434144 |
| ENSG00000168386.18 | FILIP1L | 2.474548533 | -0.877949642 | 0.000439164 |
| ENSG00000164411.11 | GJB7 | 2.524673078 | -0.976780608 | 0.000444332 |
| ENSG00000184349.12 | EFNA5 | -3.798571722 | 2.145599379 | 0.000461738 |
| ENSG00000182557.7 | SPNS3 | 1.841246073 | 1.029642088 | 0.000462008 |
| ENSG00000183570.16 | PCBP3 | 2.306916954 | -0.509721744 | 0.000465066 |
| ENSG00000226342.1 | NMD3P1 | 2.51436215 | -0.971580478 | 0.00047061 |
| ENSG00000177685.16 | CRACR2B | 1.693115294 | 2.586475022 | 0.000473899 |
| ENSG00000237976.1 | AL391069.3 | 2.736422596 | -1.307621678 | 0.000477098 |
| ENSG00000186806.5 | VSIG10L | 2.665021799 | -1.1976816 | 0.000482936 |
| ENSG00000164120.13 | HPGD | -3.009278976 | 4.312198024 | 0.000495767 |
| ENSG00000154262.12 | ABCA6 | 2.50229983 | -0.965756998 | 0.00049813 |

**Supplementary Table 2C. Differential gene expression analysis of neutrophils between our patient and healthy controls.**

| **id** | **symbol** | **logFC** | **logCPM** | **PValue** |
| --- | --- | --- | --- | --- |
| ENSG00000188672.17 | RHCE | 3.188510707 | 0.335237569 | 8.871E-12 |
| ENSG00000220267.1 | ACTBP8 | 3.396192401 | -0.521617653 | 1.26525E-10 |
| ENSG00000110203.8 | FOLR3 | 2.461826202 | 6.869668069 | 5.10368E-09 |
| ENSG00000063761.15 | ADCK1 | 2.772483308 | 0.54813153 | 9.52988E-09 |
| ENSG00000123838.10 | C4BPA | -5.740344355 | 6.149359066 | 1.13106E-08 |
| ENSG00000172322.13 | CLEC12A | -5.445782875 | 8.243355771 | 1.20842E-08 |
| ENSG00000149534.8 | MS4A2 | 3.993944193 | -1.578945608 | 1.77854E-08 |
| ENSG00000156414.18 | TDRD9 | 2.396254295 | 2.599092169 | 6.4445E-08 |
| ENSG00000004809.13 | SLC22A16 | 2.982513519 | -0.469171269 | 6.72111E-08 |
| ENSG00000111335.12 | OAS2 | -5.018582973 | 7.375992548 | 8.86449E-08 |
| ENSG00000090104.11 | RGS1 | -9.214521672 | 3.158887631 | 2.50856E-07 |
| ENSG00000172967.7 | XKR3 | 2.33723401 | 1.803984768 | 3.8215E-07 |
| ENSG00000268555.1 | AC123912.4 | -9.071040056 | 3.016419306 | 4.42299E-07 |
| ENSG00000256660.5 | CLEC12B | -8.976056239 | 2.925155859 | 6.45162E-07 |
| ENSG00000158050.4 | DUSP2 | -4.957907718 | 4.39186962 | 1.29327E-06 |
| ENSG00000160932.10 | LY6E | -4.301288011 | 7.37208198 | 1.35001E-06 |
| ENSG00000168903.8 | BTNL3 | -4.943183416 | 4.377858143 | 1.37382E-06 |
| ENSG00000089127.12 | OAS1 | -4.226574671 | 7.671477147 | 1.72033E-06 |
| ENSG00000213212.3 | NCLP1 | 4.451843451 | -1.885387489 | 2.33973E-06 |
| ENSG00000114013.15 | CD86 | -8.536206599 | 2.503053715 | 3.57808E-06 |
| ENSG00000090661.11 | CERS4 | 2.098538241 | 3.063780408 | 3.75503E-06 |
| ENSG00000196576.14 | PLXNB2 | -8.408061187 | 2.380714355 | 5.8255E-06 |
| ENSG00000196141.13 | SPATS2L | -5.576097218 | 2.844223724 | 6.03762E-06 |
| ENSG00000163993.6 | S100P | 2.00800675 | 7.234694048 | 6.25759E-06 |
| ENSG00000179639.10 | FCER1A | 2.513912057 | -0.151769333 | 6.39845E-06 |
| ENSG00000149131.15 | SERPING1 | -3.90635369 | 6.978176187 | 6.79707E-06 |
| ENSG00000120738.7 | EGR1 | -3.952031745 | 5.934535557 | 8.35359E-06 |
| ENSG00000101327.8 | PDYN | 4.130086226 | -1.837445725 | 1.12359E-05 |
| ENSG00000275713.2 | HIST1H2BH | 2.491309059 | -0.228685149 | 1.15373E-05 |
| ENSG00000140398.13 | NEIL1 | 2.698780195 | -0.637639166 | 1.18208E-05 |
| ENSG00000154269.14 | ENPP3 | 4.345730946 | -1.914966304 | 1.49481E-05 |
| ENSG00000138166.5 | DUSP5 | -8.130837353 | 2.113245893 | 1.63104E-05 |
| ENSG00000274525.1 | AC008443.7 | 2.736966388 | -0.814338745 | 1.82787E-05 |
| ENSG00000135047.14 | CTSL | -8.090977033 | 2.079766495 | 1.8985E-05 |
| ENSG00000167034.9 | NKX3-1 | -5.256096737 | 2.546457672 | 2.06423E-05 |
| ENSG00000133106.14 | EPSTI1 | -3.634647971 | 6.614891964 | 2.14301E-05 |
| ENSG00000137959.15 | IFI44L | -3.634249965 | 6.136076312 | 2.3724E-05 |
| ENSG00000128394.16 | APOBEC3F | -8.018499465 | 2.008086394 | 2.45986E-05 |
| ENSG00000253314.5 | LINC00293 | 3.56190972 | -1.60899361 | 2.5334E-05 |
| ENSG00000139044.10 | B4GALNT3 | 2.167491298 | 0.669221906 | 2.84441E-05 |
| ENSG00000166927.12 | MS4A7 | -3.822193098 | 4.245433725 | 2.95081E-05 |
| ENSG00000111331.12 | OAS3 | -3.479218111 | 8.04031182 | 3.24348E-05 |
| ENSG00000274349.4 | ZNF658 | 2.359917278 | -0.095967299 | 3.24967E-05 |
| ENSG00000187116.13 | LILRA5 | 1.874744898 | 6.407238217 | 3.37672E-05 |
| ENSG00000198574.5 | SH2D1B | -5.121835727 | 2.406567653 | 3.38266E-05 |
| ENSG00000100985.7 | MMP9 | 1.863314988 | 7.439869612 | 3.70255E-05 |
| ENSG00000106066.14 | CPVL | -4.510139257 | 2.705340718 | 3.86458E-05 |
| ENSG00000122877.15 | EGR2 | -3.96924457 | 3.669023502 | 4.27834E-05 |

**Supplementary Table 3. Fluorescently labelled antibodies used for PBMC staining.**

| **Panel 1 (for CD4 T cells)** | |  |  |
| --- | --- | --- | --- |
| **marker** | **conjugate** | **clones** | **company** |
| CD25 | FITC | M-A251 | BD |
| CXCR5 | PerCP/Cy5.5 | RF8B2 | BD |
| CD3 | PE/Cy7 | UCHT1 | BioLegend |
| CCR6 | APC | 11A9 | BD |
| CD45RA | APC/Cy7 | HI100 | BioLegend |
| CXCR3 | BV421 | 1C6/CXCR3 | BD |
| CD4 | BV510 | RPA-T4 | BD |
| CCR7 | BV711 | G043H7 | BioLegend |
| CCR10 | BUV395 | 1B5 | BD |
| CD8 | BUV805 | RPA-T8 | BD |
| CCR4 | BV605 | L291H4 | BioLegend |
|  |  |  |  |
| **Panel 2 (for Mono, NK and DCs)** | | |  |
| **marker** | **conjugate** | **clones** | **company** |
| CD14 | FITC | M5E2 | BioLegend |
| HLA-DR | PE | L243 | BioLegend |
| CD16 | PerCP/Cy5.5 | 3G8 | BioLegend |
| CD3 | PE/Cy7 | UCHT1 | BioLegend |
| CD123 | APC | AC145 | Miltenyi Biotec |
| CD56 | APC/Cy7 | HCD56 | BioLegend |
| CD11c | BV421 | B-ly6 | BD |
| CD19 | BV510 | HIB19 | BD |
| CD141 | BV605 | 1A4 | BD |
| CD15 | BV711 | W6D3 | BD |
| CD1c | BUV395 | F10/21A3 | BD |
|  |  |  |  |
| **Panel 3 (for B, CD8 T cells)** | | |  |
| **marker** | **conjugate** | **clones** | **company** |
| CD19 | BV605 | HIB19 | BioLegend |
| CD27 | FITC | O323 | BioLegend |
| CD38 | PerCP/Cy5.5 | HIT2 | BioLegend |
| CD3 | PE/Cy7 | UCHT1 | BioLegend |
| CD45RA | APC | HI100 | BioLegend |
| CD4 | APC/Cy7 | RPA-T4 | BioLegend |
| IgD | BV421 | IA6-2 | BD |
| CD8 | BV510 | RPA-T8 | BD |
| CCR7 | BV711 | G043H7 | BioLegend |

[1] M. Martin. Cutadapt Removes Adapter Sequences From High-Throughput Sequencing Reads. EMBnet.Journal; 2011, p. 10-2.

[2] A. Dobin, C. A. Davis, F. Schlesinger, J. Drenkow, C. Zaleski, S. Jha *et al.* STAR: ultrafast universal RNA-seq aligner. Bioinformatics, 2013;29**:**15-21.

[3] S. Anders, P. T. Pyl, W. Huber. HTSeq--a Python framework to work with high-throughput sequencing data. Bioinformatics, 2015;31**:**166-9.

[4] M. Ota, Y. Nagafuchi, H. Hatano, K. Ishigaki, C. Terao, Y. Takeshima *et al.* Dynamic landscape of immune cell-specific gene regulation in immune-mediated diseases. Cell, 2021;184**:**3006-21.e17.

[5] M. D. Robinson, D. J. McCarthy, G. K. Smyth. edgeR: a Bioconductor package for differential expression analysis of digital gene expression data. Bioinformatics, 2010;26**:**139-40.

[6] T. Wu, E. Hu, S. Xu, M. Chen, P. Guo, Z. Dai *et al.* clusterProfiler 4.0: A universal enrichment tool for interpreting omics data. Innovation (N Y), 2021;2**:**100141.

[7] O. Solomon, V. Kunik, A. Simon, N. Kol, O. Barel, A. Lev *et al.* G23D: Online tool for mapping and visualization of genomic variants on 3D protein structures. BMC Genomics, 2016;17**:**681.
