# Supplementary figures and images for: A novel functional IKBKE variant activating NFAT in a patient with polyarthritis and a remittent fever
Source: Front Immunol. 2024 Oct 25;15:1475179. doi: 10.3389/fimmu.2024.1475179 (PMC11544129; doi:10.3389/fimmu.2024.1475179)

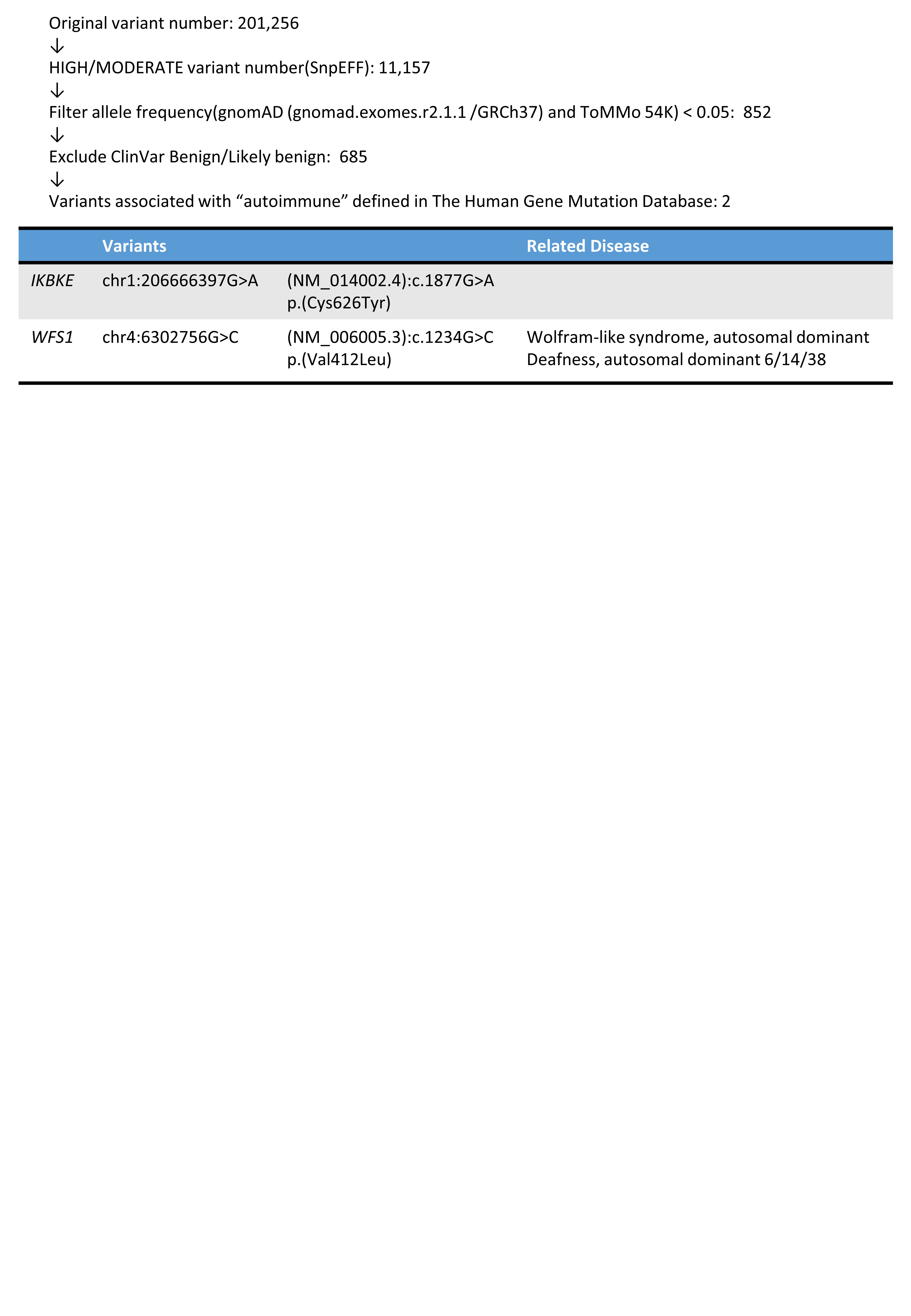

Supplement: Supplementary file 2 [file Image1.jpeg]

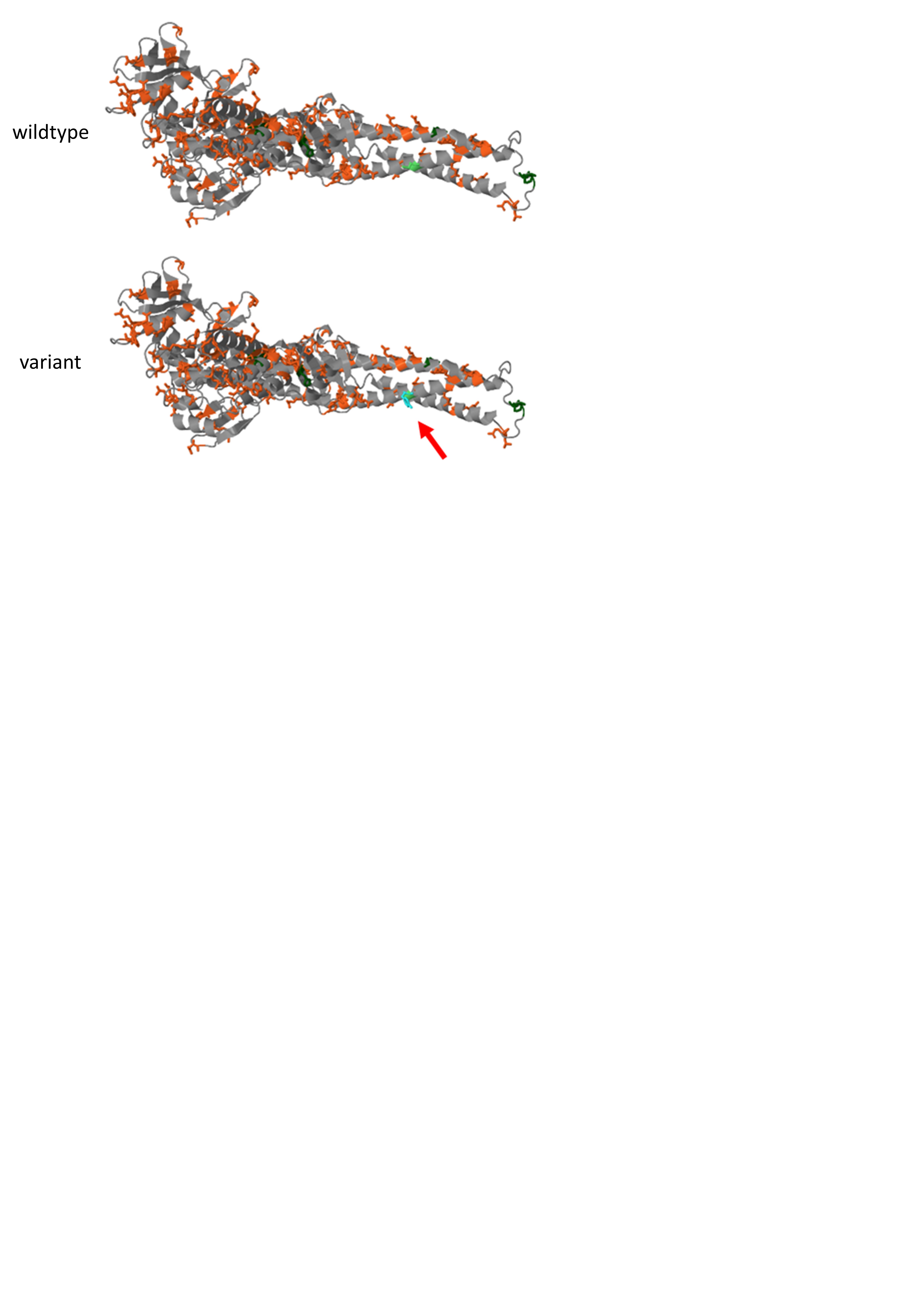

Supplement: Supplementary file 3 [file Image2.jpeg]

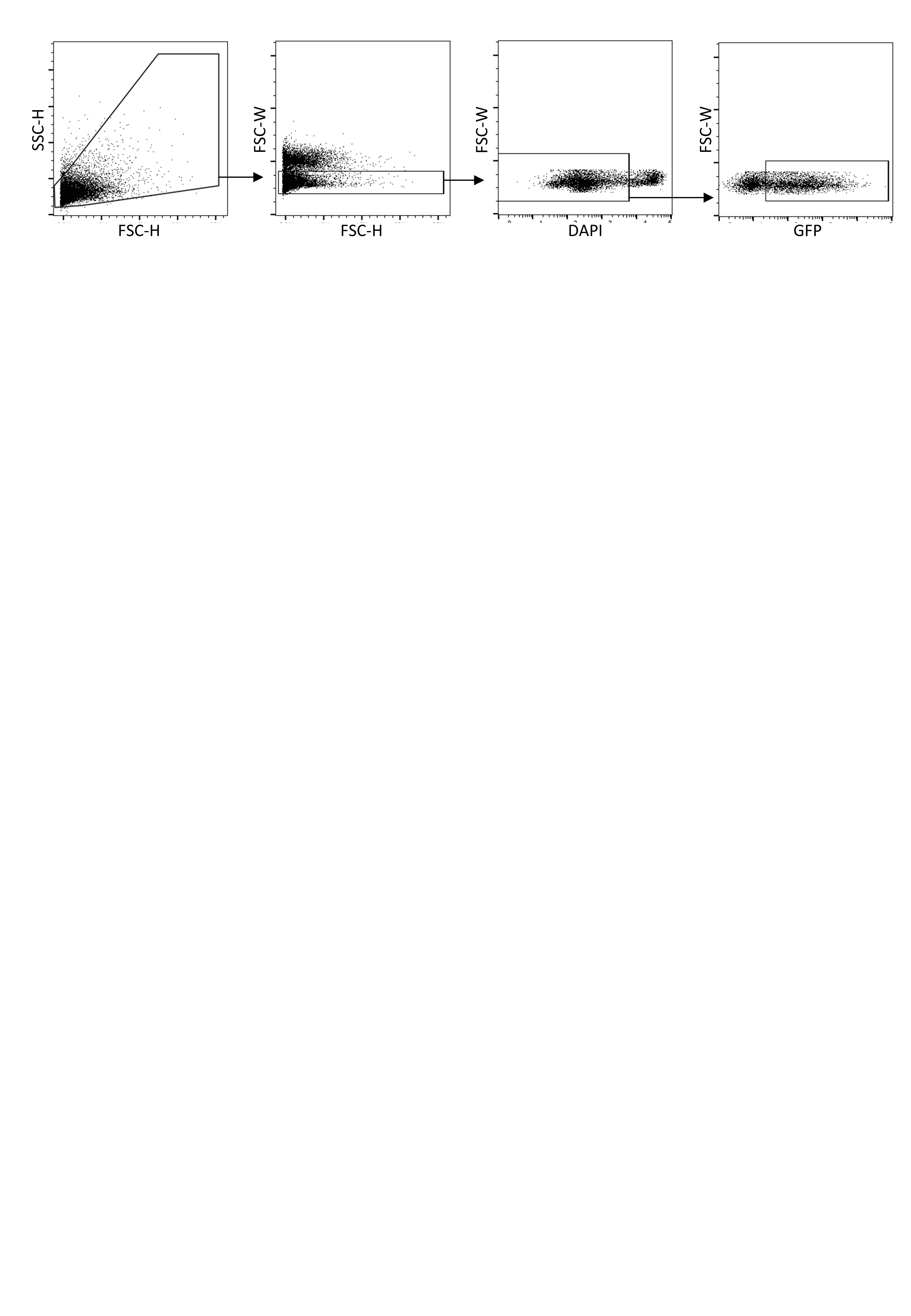

Supplement: Supplementary file 4 [file Image3.jpeg]
